# Formation of Condition‐Dependent Alpha‐Synuclein Fibril Strain in Artificial Cerebrospinal Fluid

**DOI:** 10.1002/advs.202505228

**Published:** 2025-11-20

**Authors:** Rūta Sniečkutė, Darius Šulskis, Arūnė Jocytė, Urtė Venclovaitė, Rimgailė Tamulytė, Mantas Žiaunys, Vytautas Smirnovas, Andrius Sakalauskas

**Affiliations:** ^1^ Institute of Biotechnology Life Sciences Center Vilnius University Sauletekio al. 7 Vilnius LT‐10257 Lithuania; ^2^ Institute of Biochemistry Life Sciences Center Vilnius University Sauletekio al. 7 Vilnius LT‐10257 Lithuania

**Keywords:** aggregate structure analysis, alpha‐synuclein aggregation, Cryo‐EM, Parkinson's disease, physiological conditions

## Abstract

α‐Synuclein (aSyn) is an intrinsically disordered protein involved in neurotransmission and synaptic plasticity. The pathological aggregation of this protein is a hallmark of synucleinopathies such as Parkinson's disease (PD) or Multiple System Atrophy (MSA). Misfolded aSyn, which primarily originates in the cell cytosol, transmits between neurons, promoting a prion‐like propagation. However, extracellular environments such as interstitial and cerebrospinal fluids (ISF & CSF) play a major role in its clearance and pathological transformation. The molecular components of CSF, including proteins, glycosaminoglycans, and metal ions, may influence the aggregate morphology, structure, and cytotoxicity to cells. To better understand how extracellular composition affects aggregates and their formation, artificial cerebrospinal fluid (aCSF) is employed to mimic potential aggregation processes occurring in CSF. Distinct aSyn fibrils are observed that exhibited low stability outside aCSF, and the removal of key CSF components led to its structural alterations. Cryo‐electron microscopy revealed that these fibrils possess an electron density pocket coordinated with polar basic AAs (K43, K45, H50) that is also observed in aggregates obtained from PD and MSA patients. The findings illustrate the importance of physiologically relevant conditions in studying aSyn aggregation and may explain why disease‐related fibril structure replication in vitro has not yet been successful.

## Graphical abstract

α‐synuclein aggregation in artificial cerebrospinal fluid (aCSF) leads to a distinct conformation with an electron density pocket motif found in aggregates from PD and MSA patients. Such fibrils has low stability outside the reaction conditions, hinting about the influence of cerebrospinal fluid components not only on the formation, but also on the stability of α‐synuclein aggregates.

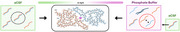

## 1 Introduction

α‐Synuclein (aSyn) is a protein primarily found in the brain, playing a crucial role in neuronal functions such as synaptic vesicle regulation and neurotransmitter release.^[^ ^1^, ^2^ ^]^ Its abnormal aggregation is a hallmark of several neurodegenerative diseases, including Parkinson's disease (PD), dementia with Lewy bodies, and other synucleinopathies.^[^ ^3^, ^4^ ^]^ In these pathologies, aSyn misfolds and forms insoluble aggregates in neurons that are called Lewy bodies and Lewy neurites.^[^ ^5^ ^]^ Accumulation of abnormal aggregates leads to impaired neurotransmitter release, mitochondrial dysfunction, loss of presynaptic proteins, and disruption of protein trafficking that generally results in neuronal loss and neurodegeneration.^[^ ^6^ ^]^

One of the key aspects of aSyn is its ability to propagate between cells via different pathways^[^ ^7^, ^8^ ^]^ and in diverse pathological forms – soluble oligomers and insoluble aggregates. aSyn and its soluble misfolded species are mostly contained within the cell; however, there is evidence that it may spread by diffusion to the intercellular space.^[^ ^9^ ^]^ This can occur via (1) direct translocation across the membrane that is facilitated by a specific protein such as DnaJ homolog subfamily C member 5 (DNAJC5),^[^ ^10^ ^]^ (2) by calcium‐mediated exosomal secretion^[^ ^11^, ^12^ ^]^ or other neuronal activity‐dependent pathways.^[^ ^13^ ^]^ Soluble aSyn species that escaped the cell can be absorbed by other neurons, mostly through endocytosis and passive diffusion across the plasma membrane.^[^ ^14^, ^15^ ^]^ However, aSyn aggregates formed within the cell can be secreted from the neuron, enabling the interaction with the neighboring neurons or immune cells, which can uptake them. This process contributes to spreading the pathology across brain regions following a prion‐like mechanism.^[^ ^16^, ^17^ ^]^ The aggregate transmission may occur through exocytosis and endocytosis, or through tunnelling nanotubes for larger assemblies.^[^ ^18^ ^]^ Once inside the recipient neuron, the misfolded aSyn induces native aSyn to misfold and aggregate, perpetuating a cycle of toxic accumulation leading to cell death and progression of neurodegeneration.^[^ ^19^, ^20^ ^]^

While aSyn aggregation is considered to originate within the neuron at presynaptic terminals,^[^ ^21^, ^22^ ^]^ different transport pathways of this protein and its misfolded species through synapses play a crucial role in disease development. The neuron environment, consisting of interstitial and cerebrospinal fluids (ISF & CSF), is crucial for maintaining the brain's homeostasis and ensuring proper neuronal function.^[^ ^23^, ^24^ ^]^ These fluids are part of the brain's microenvironment, and their interaction is important for waste removal, delivery of nutrients, and overall brain health.^[^ ^25^ ^]^ In the case of disease pathology, the extracellular space contains various forms of the protein, from native aSyn to toxic misfolded species.^[^ ^26^ ^]^ Here, the constant exchange between ISF and CSF plays a major role in clearing aSyn from the brain, ^[^ ^27^ ^]^ highlighting the importance of their interaction in the context of neurodegenerative diseases.^[^ ^28^ ^]^

The misfolded aSyn deposits within the extracellular space may change, seed the aggregation of cell‐free aSyn,^[^ ^29^ ^]^ and result in various fibril conformations due to shifted environmental conditions.^[^ ^30^, ^31^ ^]^ Differences in crowding, ionic strength, and reaction component composition play a crucial role in the formation of both *de novo* and seeded aggregates.^[^ ^32^, ^33^ ^]^ There is evidence that glycosaminoglycans (such as heparin), ^[^ ^34^ ^]^ metal ions,^[^ ^32^ ^]^ lipoproteins,^[^ ^35^ ^]^ or other proteins such as human serum albumin^[^ ^36^ ^]^ shape the formation of aSyn fibrils, resulting in different aggregate structures. This is why it is no surprise that multiple distinct aSyn aggregate structures formed in vitro and in vivo were recorded using cryo‐electron microscopy, ^[^ ^37^, ^38^, ^39^, ^40^ ^]^ with cases describing unknown molecules within the core of the aggregate.^[^ ^41^, ^42^ ^]^ Such residual molecules from either cellular or extracellular environments suggest the importance of studying the aggregation of aSyn under physiologically relevant conditions.

The study of aSyn fibril formation in the extracellular environment may provide critical insights into the development of aSyn related neurodegenerative diseases. While it was not yet possible to fully template the disease‐derived fibril structure using recombinant aSyn protein under in vitro conditions,^[^ ^41^ ^]^ the search for a model system that would help in simplifying the templating process and improving our understanding of disease‐related mechanisms is very important. In this research, we have used artificial cerebrospinal fluid (aCSF) that contains major CSF components to simulate the aggregation of aSyn in the extracellular environment. The aCSF conditions yielded a distinct aggregate conformation with a low stability outside the aCSF environment, while removal of major cerebrospinal fluid components resulted in structural variation between the formed aggregates. Cryo‐electron microscopy (Cryo‐EM) imaging analysis showed a 3D structure with a small molecule entrapped within the fibril core. These results demonstrate that mimicking the CSF environment is essential for retaining aSyn aggregate structures formed within aCSF, which may lead to successful replication of ex vivo aggregates in vitro.

## 2 Results

### 2.1 aCSF Components Stabilize the Aggregation Process of aSyn

**Figure 1:**
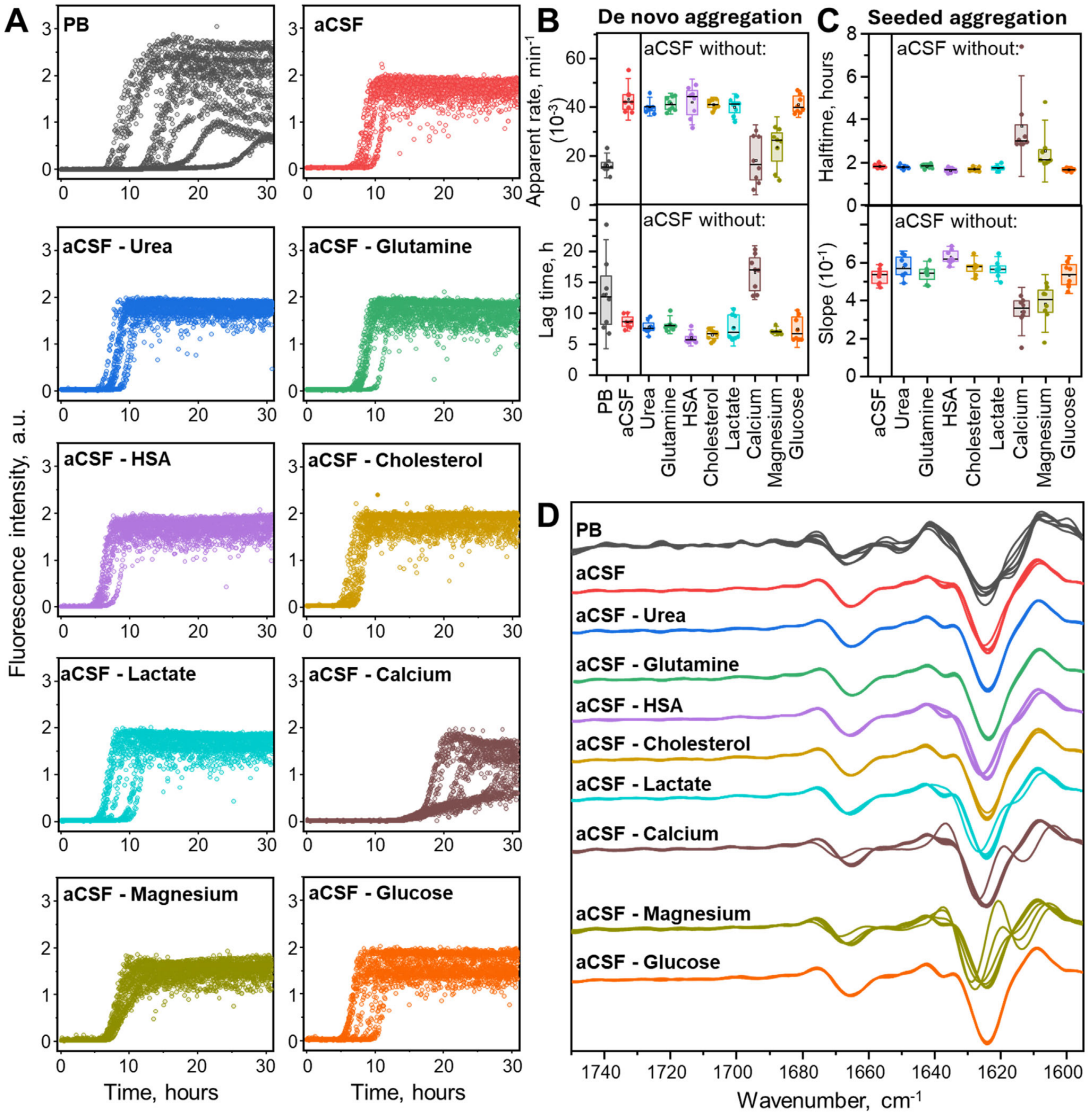
Influence of aCSF composition on aSyn aggregation kinetics and fibril secondary structure. Aggregation kinetics in different aCSF compositions (A) and its parameters of de novo (B) and seeded (C) fibrillation process. The analysis of aggregation kinetic parameters provides quantitative information about the robustness of the aggregation process, where low repeatability (higher variations) between technical repeats signifies potentially different aggregation pathways that may lead to the formation of different aggregate types. The most variable data was found to be in PB and aCSF without calcium/magnesium conditions. Resulting aSyn aggregate second derivative FTIR spectra (D) are overlaid based on aCSF composition where aSyn was aggregated. FTIR second derivative provides information about average differences between sample reads, indicating absolute secondary structure differences between the samples. Each condition is represented with separate individual technical repeats (n = 8), the box plot is of 25 – 75 % range, the whisker range is 1.5 SD, line within the box represents the median.

To understand the influence of formulated artificial cerebrospinal fluid (aCSF) on the aSyn aggregation process, we have designed kinetic experiments in phosphate buffer (*further referred as* – PB (A component of aCSF)), aCSF, and solutions with varying aCSF components (**Figure** **1**A). Since aSyn is prone to polymorphism under certain sets of conditions,^[^ ^43^ ^]^ we evaluated the molecules within aCSF that can modify or stabilize the aSyn aggregation pathway. In order to assess the variability of aSyn aggregation, eight replicates for each condition were used, as well as an additional 8 technical repeats in PB and aCSF conditions using a different aSyn purification batch (Figure S1, Supporting Information). Out of all ten variations displayed, the most contrastive conditions were PB, without calcium and without magnesium, which resulted in more scattered *de novo* aggregation kinetic parameters (Figure 1B). PB environment exhibited the most variation in aggregation lag time, but the deviation in the apparent rate constant was among the lowest. Calcium played a significant role in increasing the overall aggregation speed, whereas magnesium only affected the apparent rate constant.

The kinetic data variation also translated to seeded aggregation (Figure 1C; Figure S2, Supporting Information) and the resulting fibril second derivative FTIR spectra (Figure 1D). The removal of calcium and magnesium resulted in slower seeded aggregation kinetics and different FTIR spectra among the technical repeats. While it is known that calcium promotes aSyn aggregation by binding to C terminal of this intrinsically disordered protein,^[^ ^44^ ^]^ the synergistic effect of other aCSF components (including magnesium) favours the formation of aggregates with distinct FTIR spectra second derivatives. Unlike the spectra recorded for most of the aggregates formed in aCSF conditions (including its variations), PB aggregate FTIR second derivatives showed an additional prominent minimum at 1616 – 1618 cm^−1^, indicating a stronger β‐sheet region. The removal of Ca^2+^ or Mg^2+^ ions resulted in a few specific aggregate spectra exhibiting minima at 1615 – 1613, and 1627 – 1629 cm^−1^, which may also correspond to the formation of an aSyn aggregate type with different beta‐sheet regions and stabilized orientation.^[^ ^45^ ^]^ To confirm data variability, another batch of aSyn was aggregated in above mentioned reaction mixtures (Figure S3, Supporting Information). The residual aSyn, evaluated from SDS‐PAGE images (Figure S4, Supporting Information), endpoint maximum fluorescence intensity, and sample optical density, confirms the highest data disparity in the PB reaction mixture, and aCSF conditions without Ca^2+^ and Mg^2+^. Additionally, the maximum Thioflavin‐T (ThT fluorescence excitation‐emission positions and corresponding intensities were recorded, which were in line with previous observations. This data variability may result in different ThT interactions with the fibril's surface that can correlate with different aggregate structural motifs.

### 2.2 aCSF Fibrils are More Homogeneous than Those Produced in PB

**Figure 2:**
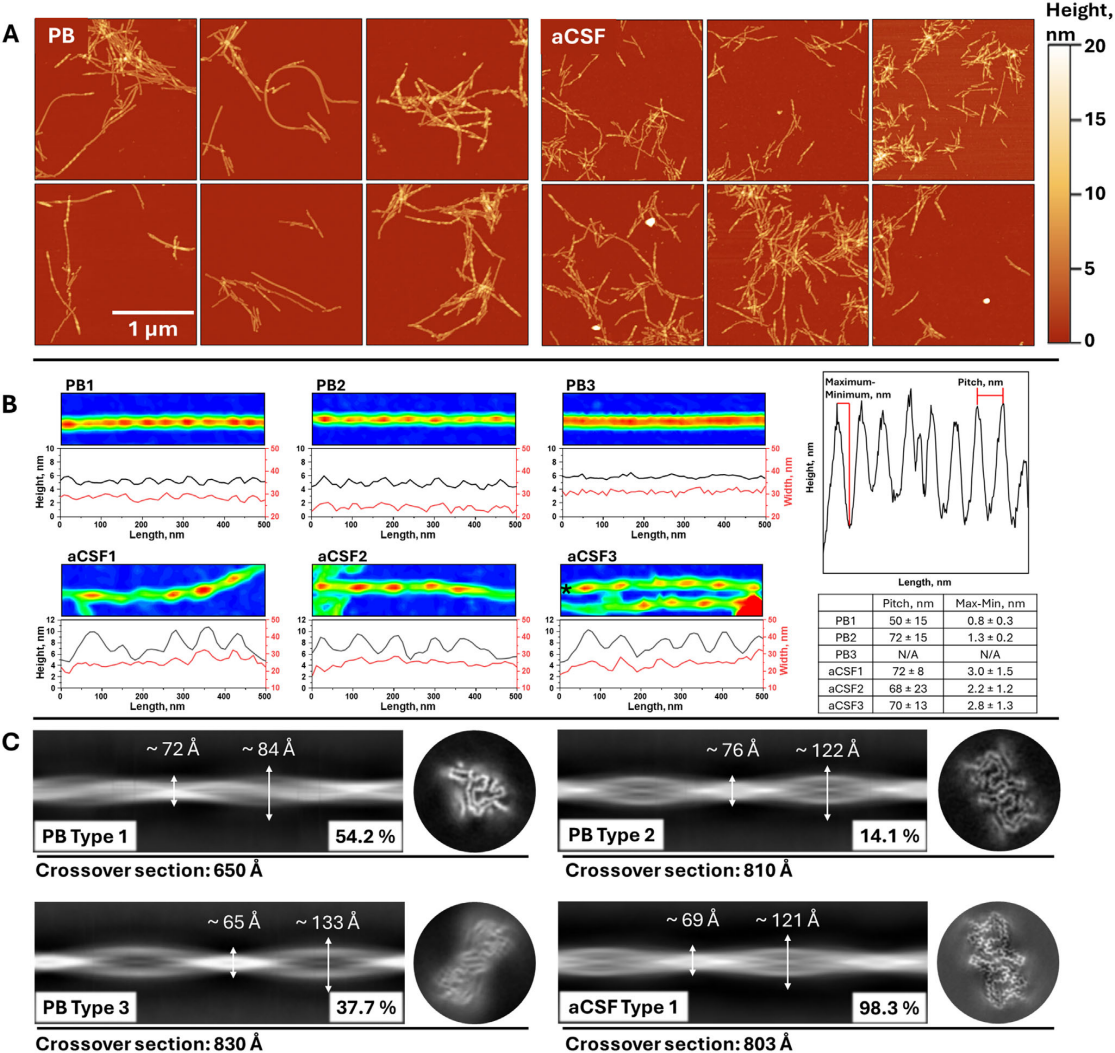
End products of aSyn aggregation recorded using AFM (A) and fibril morphology examples (B) that were formed in PB or aCSF. Cropped 2.5 µm x 2.5 µm images of the original 10 µm x 10 µm are shown in order to provide a clear view of different positions on the mica. The different structural polymorphs are readily visible at the displayed heatmaps for each example AFM image with fibril height and width traces. In aCSF3, * indicates analysed filament. Pitch and Max‐Min averages across the fibril are summarized in the adjacent table. A clear difference between PB type fibrils is visible, where PB3 does not contain expressed twist periodicity. Distinct fibril types observed in PB and aCSF using Cryo‐EM (C), along with their probability and crossover section of the 3D map. Each 2D class projection was analyzed using Fiji software to extract the full FWHM at the widest and narrowest positions for comparison. The cross‐section maps do not represent equal scaling.

To investigate the differences between the end‐products of aSyn aggregation in PB and aCSF, Atomic force microscopy (AFM) and Cryo‐EM imaging were employed (**Figure** **2**). Due to the large quantity of samples after kinetic experiments, AFM images were taken from the samples that had distinct FTIR spectra among its group. Fibrils found in PB appeared longer and more widely dispersed than those displayed in aCSF (Figure 2A; Figure S5, Supporting Information). Additionally, in PB multiple distinct species of fibrils were observed (Figure 2B). These fibrils possessed different pitches and periodicity height variation (Max‐Min). PB1 and PB2 had no significant pitch difference but contrasted by periodicity height. PB3 fibrils displayed little to no periodicity or low variations, potentially due to the lower AFM resolution that was used. In contrast, aCSF fibrils were clustered, reducing the proficiency of such evaluation; however, all selected fibrils were uniform in pitch and Max‐Min of the periodicity. To reduce the effect of AFM sample preparation (APTES‐functionalized mica and sample wash procedures), cryogenic electron microscopy was used (Figure 2C; Figures S6–S9, Supporting Information). The Cryo‐EM images confirmed similar tendencies as observed in AFM. The results revealed three distinct fibril species in PB, compared to one dominant type in aCSF. Additionally, PB type 1 had a twist Max‐Min difference of ≈12 Å. Based on the obtained structure micrographs, PB type 1 resembles one filament of PB type 2 that is seemingly similar to aCSF Type 1 structure (Figure S10, Supporting Information).

### 2.3 aSyn Fibrils form a Distinct Structure in aCSF

**Figure 3:**
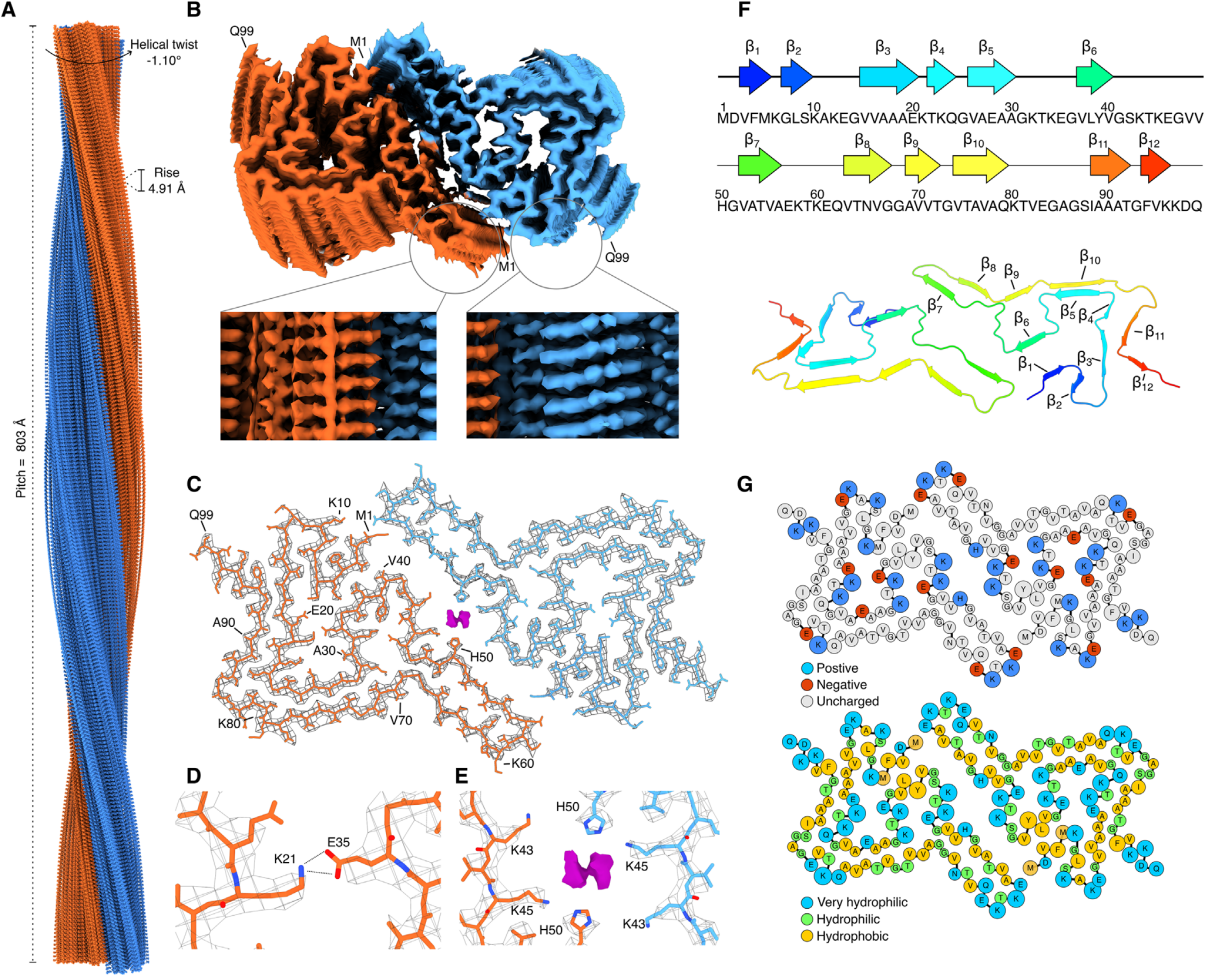
Cryo‐EM model of aSyn fibrils formed in aCSF. Side view (A) and top view (B) of the reconstructed Cryo‐EM map. Single cross‐section of the atomic model (residues 1‐99) built in the density map (C). The salt bridge (D) is formed between K21 and E35 residues. Ligand density embedded inside the fibril core (E). The red and blue colors represent oxygen and nitrogen atoms, correspondingly, while orange/cyan display the carbon backbone. β‐sheet arrangements in the structure (F). Charge and hydrophobicity/hydrophilicity distributions (G) in the structure, where blue, green, and yellow colors represent very hydrophilic, hydrophilic, and hydrophobic amino acids, correspondingly.

Cryo‐EM was used to solve the structure of aSyn fibrils formed in aCSF. The spontaneous aSyn fibril formation was in the presence of ThT, however, the samples used for Cryo‐EM were reseeded without adding this amyloidophilic dye. The fibrils contained two intertwined protofilaments that exhibited a C2 symmetry with a pitch of 803 Å, helical twist –1.10°, and a helical rise of 4.91 Å (**Figure** **3**A,B). During the map building, pseudo‐2_1_‐screw axis symmetry was also tested, but it led to a lower resolution map compared to C2, and β‐sheets did not separate. The 3D reconstructed map extended from 1 to 99 residues, covering both N‐terminal and entire non‐amyloid beta component (NAC) regions, with the estimated final resolution of 2.9 Å (Figure 3C). However volume map of aCSF fibrils at a lower threshold level indicates extra density, suggesting a β‐sheet from the C‐terminal (Figure S11, Supporting Information). One salt bridge between lysine 21 and glutamine 35 was identified via ProteinTools webserver^[^ ^46^ ^]^ (Figure 3D) and ligand embedded within the fibril core (Figure 3E). The ligand binding pocket was coordinated between K43, K45, and H50 residues, with potential glucose, glutamine, various metal‐ion or salt‐water complexes from aCSF fitting the electron density. Similar pocket and electron densities were previously observed in filaments extracted from patients with multiple system atrophy (MSA)(Figure S12, Supporting Information).^[^ ^47^ ^]^ Twelve parallel β‐sheets were observed in the secondary structure, with β5 and β10 forming steric zipper motifs (Figure 3F). We also analyzed the distribution of charges and hydrophilicity in the solved structure (Figure 3G). The positive charges were clustered in the ligand‐binding core and along the fibril's side, arranged by K10, K12, K58, and K60 lysines, which could allow the binding of negative charge molecules like DNA. Hydrophilic sites were also located at the same positions, whereas most hydrophobic space was between β1 and β6 sheets. The overall structural isomorph did not resemble previously solved structures in CSF,^[^ ^26^ ^]^ however, they were comparable with polymorphs 6b from PD patient (PD405) and 6c from MSA patients (MSA363 & MSA333) described by Burger et al.^[^ ^48^ ^]^ Moreover, comparison of one aCSF protofilament with other already published structures reveals only partial similarities, with the most related being Tyr39 aSyn fibrils^[^ ^49^ ^]^ (PDB:6L1T) and apo WT polymorph 5a^[^ ^50^ ^]^ (PDB: 8ZLP) (Figure S13, Supporting Information).^[^ ^47^, ^49^, ^50^, ^51^, ^52^ ^]^

### 2.4 Structural integrity of aCSF fibrils

**Figure 4:**
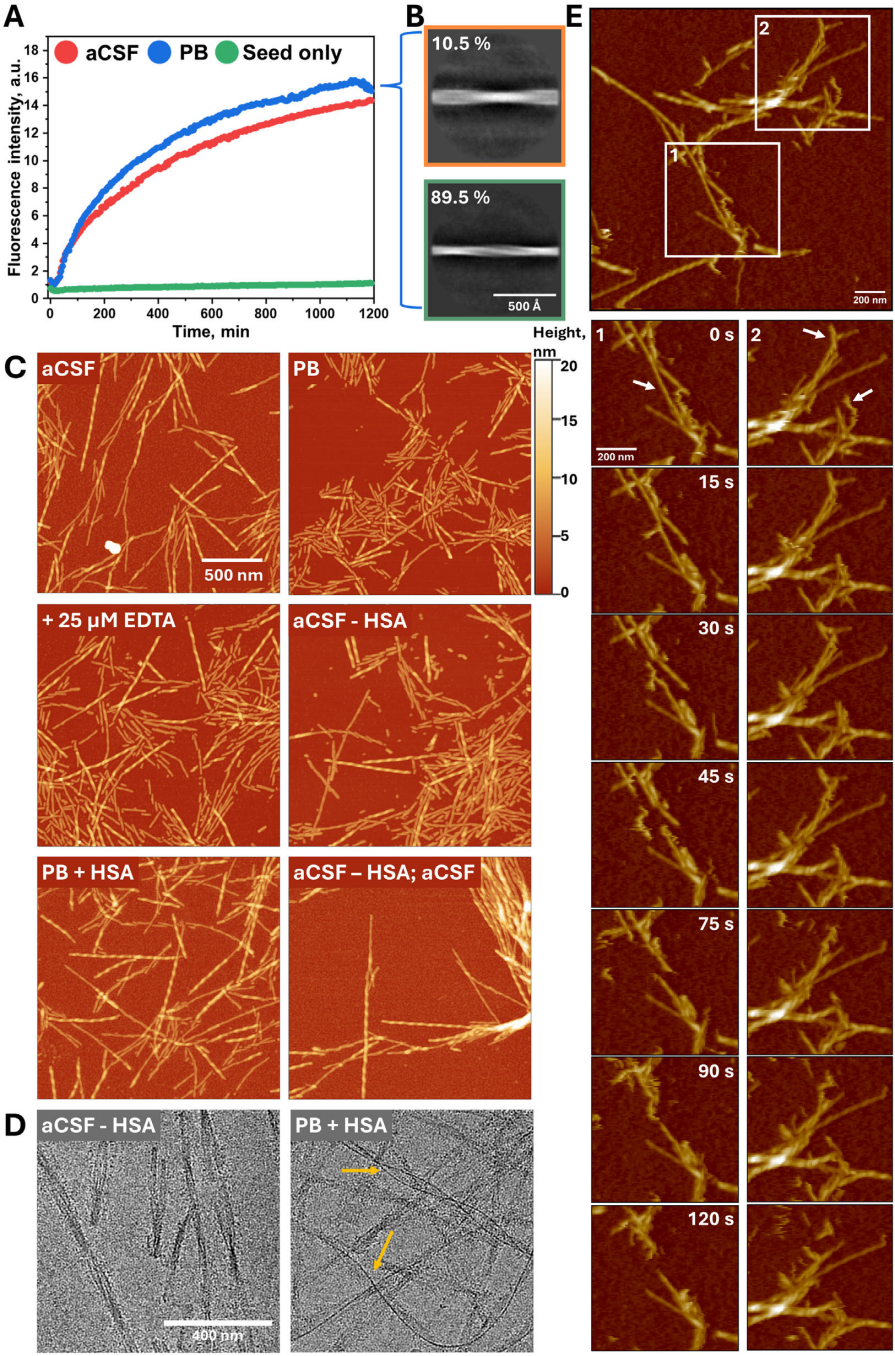
aCSF fibril seeding process in aCSF and PB solutions (A) and the resulting aggregate composition formed in PB solution, characterized using Cryo‐EM (B). No significant difference between endpoint ThT fluorescence intensity was observed, while 2D cryo‐EM displays ≈1:9 ratio of two distinct fibril conformations. The resulting AFM images (C) of aCSF fibrils 10 min after resuspension in different aCSF solution compositions (indicated in the top left corner) and Cryo‐EM images (D) after incubating the samples for 24 h. The aCSF–HSA / aCSF indicates the fibrils resuspended in aCSF–HSA solution and then, after 10 min incubation, resuspended in aCSF. aCSF fibril imaging in PB solution on a mica using HS‐AFM (E).

To understand whether the fibrils formed in the aCSF template their structure, a seeded aggregation kinetics experiment was performed using sonicated preformed fibrils in aCSF and PB (**Figure** **4**A), yielding statistically similar endpoint ThT fluorescence intensity values (Figure S14, Supporting Information). The residual concentration of soluble (non‐aggregated) aSyn was comparatively larger when the seeding experiment was done in the aCSF solution (Figure S15, Supporting Information). This suggests that the templating process does not produce identical aggregates to the ones used for seeding. Therefore, the resulting aCSF fibrils seeded in PB solution were characterized using Cryo‐EM (Figure 4B; Figure S8, Supporting Information), which showed two distinct fibril conformations (ratio of≈1:9). To understand this process further, seeds were resuspended in PB and aCSF solutions (including its variation without specific components or with the addition of 25 µM of EDTA) and imaged using AFM (Figure 4C; Figures S16 and S17A, Supporting Information). Based on the visual inspection of fibrils on the mica, the removal of human serum albumin (HSA) from the aCSF fibrils solution or the addition of EDTA affected the aggregate morphology, while the removal of other aCSF components showed similar morphologies to the original aCSF fibrils. These fibrils without HSA were mostly shorter than the ones found in the control sample. Nonetheless, such fibrils are not found on the mica once the sample is resuspended back to solution with HSA (aCSF).

To further investigate the impact of HSA removal from aCSF fibril solution, we incubated fibrils in the corresponding solutions for 24 h and performed Cryo‐EM (Figure 4D; Figure S17B, Supporting Information). Unlike in AFM images, visually observed fibrils of the frozen hydrated micrographs were of similar length. However, the aggregates incubated in the solution with HSA had long, thin rod‐like structures alongside the fibril's axis (white arrows), with a couple of cases where two such structures are bound to the fibril, indicating potential HSA interaction along the axis of fibrils. However, the SDS‐PAGE analysis did not confirm the stable interaction between the fibrils and HSA (Figures S4 and S18, Supporting Information), because the supernatant of pelleted fibrils contained the HSA concentration that was similar to the initial reaction mixture's. Concerning the probable effect of sample preparation (centrifugation, binding on a functionalized mica, freezing), we performed HS‐AFM (Figure 4E; Videos S1 and S2, Supporting Information), where removal of HSA was done after the fibrils were absorbed on the APTES‐functionalized mica. The rapid aggregate separation was observed within 10 min after the change of solution, while control conditions did not show any significant changes (Videos S3 and S4, Supporting Information). These results confirmed the restructurization of aCSF fibrils in an HSA‐absent environment. However, the excitation‐emission matrix (EEM) scans (Figure S19, Supporting Information) that can be used to identify different binding modes of ThT to fibrils showed no notable variation in EEM intensity maximum positions throughout the test. This means that even though fibrils restructure, their binding mode to ThT does not change significantly.

### 2.5 aSyn Fibrils from aCSF Exhibit Enhanced Toxicity

**Figure 5:**
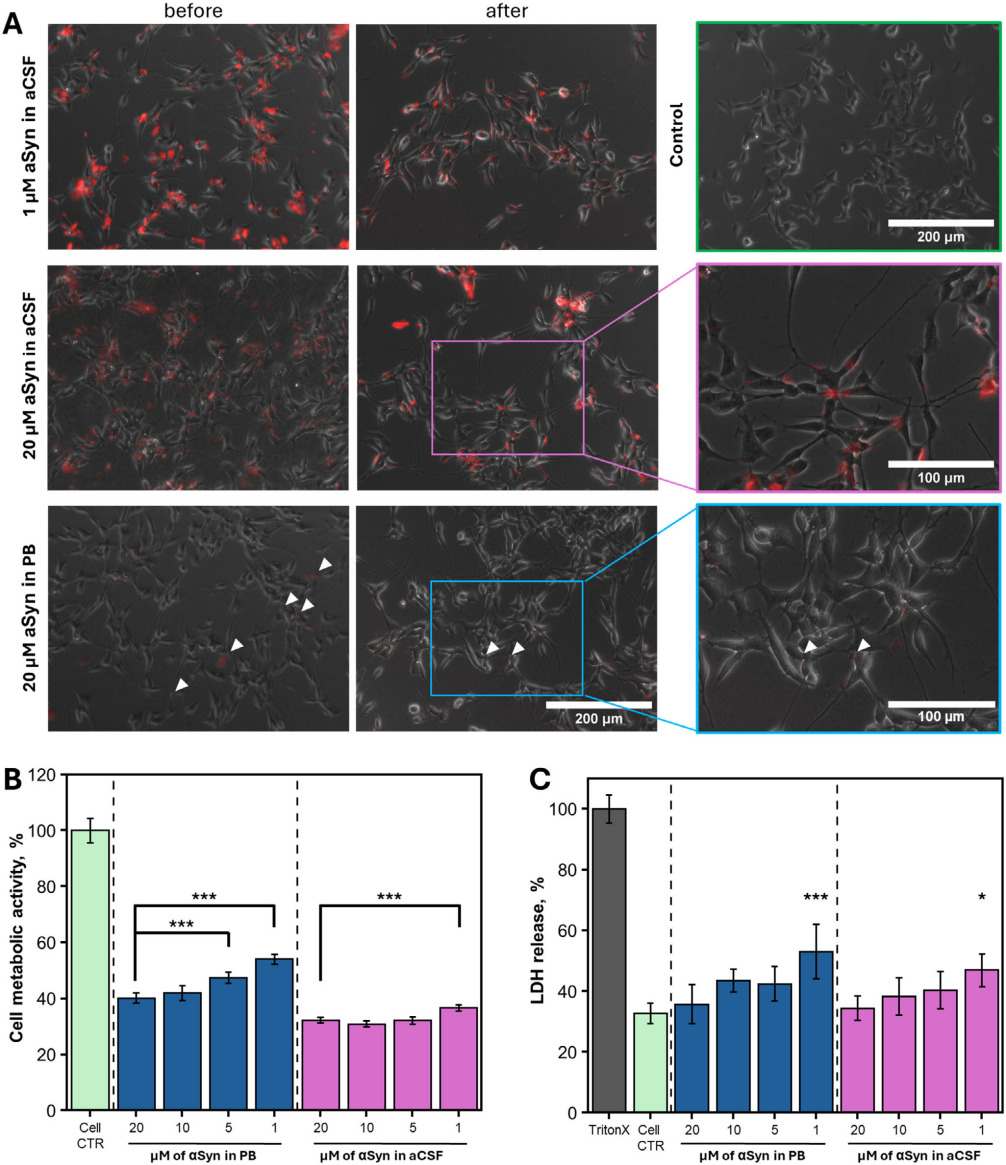
Effect of aSyn aggregates formed in PB or aCSF on SH‐SY5Y human neuroblastoma cells. (A) EVOS microscopy images of cells treated with 1 and 20 µm of aSyn aggregates formed in aCSF or PB (the aggregation was performed with the labelled aSyn in a ratio of 95% aSyn, 5% mCherry‐aSyn). Untreated SH‐SY5Y cells were used as a control. Images were collected before and after the media change. Changes in cell metabolic activity (B) and cytotoxicity (C) after incubation with different types of aSyn aggregates in concentration dependent manner. One‐way ANOVA Bonferroni means comparison (n = 9) for each condition was conducted to determine differences between sample concentrations (*p < 0.05, **p < 0.01, ***p < 0.001). For LDH release, the comparison was with the cell control. Error bars show standard deviation (n = 9).

Finally, the sample evaluation on the cellular level was conducted (**Figure** **5**). The SH‐SY5Y human neuroblastoma cell line was used to determine whether aSyn aggregation products formed in aCSF or PB solutions possessed distinct effects. The MTT assay was applied for cellular metabolic activity measurement, where it indicated potent viability reduction and aggregate type dependent differences (Figure 5B). Cells treated with aSyn aggregates formed in PB had significantly higher viability compared to ones of aCSF (Table S1, Supporting Information). Here, lower concentrations of fibrils resulted in increased metabolic activity, while the titration of aSyn aggregates formed in aCSF showed an invariable effect. Meanwhile, the LDH release assay revealed that these two types of aggregation products possessed a similar effect on the cell membrane, with the most toxic concentration being 1 µm (Figure 5C). Additionally, cell imaging was conducted to visualize the distribution of aSyn aggregates and possible interaction with SH‐SY5Y cells. For such evaluation, the microscopy data were collected before and after cells were washed and the media was changed to one without the aggregates (Figure 5A and Experimental Section). Despite the equal concentration added, aSyn aggregates formed in aCSF had a more active fluorescence signal than the aggregates from PB. After overnight incubation, aSyn aggregates from PB tended to assemble into several individual clusters (Figure 5A, white arrowheads) while other types of aggregates were distributed throughout the cell culture by forming a monolayer. However, the wash‐out and media change that potentially had to remove all unbound aggregates from the measurement cell revealed that aggregation products formed in aCSF had a higher affinity to SH‐SY5Y cells compared to fibrils originated in PB.

## 3 Discussion

Growing knowledge in the field of synucleinopathies, including Parkinson's disease, has indicated the role of aSyn aggregation as a critical driver of disease progression and heterogeneity.^[^ ^53^ ^]^ A key focus has been on the protein's properties to adopt distinct conformations and propagate in a prion‐like manner. However, recent evidence^[^ ^48^, ^54^, ^55^, ^56^ ^]^ shows that aSyn extracted from brain tissue or cerebrospinal fluid fails to completely replicate its structure to the recombinant protein in vitro, resulting in the partial templating of its ternary assembly.^[^ ^40^, ^54^ ^]^ These findings shift the research to a new direction, where the native aggregation environment in the brain tissue, cells, or CSF may play a significant role in determining fibril formation, structure, and morphology. These differences raise the importance of studying aSyn aggregation in physiologically relevant conditions. To address this point of view, we used artificial cerebrospinal fluid to recreate the environment closer to in vivo conditions where aSyn aggregates formation and templating processes occur.

Our findings demonstrate that components from artificial cerebrospinal fluid dictate the formation of a specific aSyn fibril structure. As expected, divalent metal ions (Ca^2+^ and Mg^2+^) emerged as key modulators of protein aggregation kinetics and structural variation observed using FTIR. While calcium and magnesium are well known to interact with the negatively charged C‐terminal region of aSyn,^[^ ^57^, ^58^ ^]^ the aCSF formulation includes HSA that chelates Me^2+^ and is proven to increase its interaction with aSyn N‐ and C‐terminals once Cu^2+^ ions are bound to it.^[^ ^59^, ^60^ ^]^ It is probable that such interaction specifically occurs in our case, as the removal of HSA or the addition of EDTA (to bind all divalent metals) resulted in the fragmentation of preformed aCSF fibrils and failure to 100% replicate its conformation. This suggests that fibrils produced in aCSF are dynamic when the critical components that stabilize their structure are removed, which contradicts the common belief that once fibrils form, they retain a fixed structure due to its energy minimum.^[^ ^31^ ^]^ It is worth noting that similar structure fibrils are formed in PB conditions (PB Type 2). Such fibrils share a non‐dominant part of structures in the sample; however seemingly are made of two PB Type 1 filaments (Figure S10, Supporting Information). This suggests that reseeded aCSF fibrils in PB lead to a 1:9 ratio between two‐filament and one‐filament fibrils, while *de novo* formed aCSF fibrils have a dominant two‐filament structure (Figure 3; Figures S6 and S9, Supporting Information). This shift in assembly may be directed by the interface of aCSF components such as Me^2+^ ions and HSA. Fibril morphological instability complicates matters for in vitro research due to sample preparation limitations (fibril resuspension in measurement buffer solutions, extraction method from ex vivo samples, desalting procedures, protein misfolding cyclic amplification (PMCA), and many other factors).^[^ ^26^, ^40^, ^48^, ^61^, ^62^ ^]^ It could be that ex vivo fibrils transform, resulting in a different morphology or filament assembly once observed using AFM, Cryo‐EM, or other methods. This environment‐specific shift may also explain why it is possible to replicate fibril filaments, but rather not possible to replicate the full structural assembly of extracted fibrils in vitro.^[^ ^40^, ^48^, ^54^, ^55^, ^56^ ^]^

We also determined the Cryo‐EM structure of aCSF fibrils, which does not have an exact match with any currently published structure (Figure S13, Supporting Information). However, Burger and his colleagues^[^ ^48^ ^]^ show similar fibril conformation extracted from PD (6b) and MSA patients (6c), which yield a different filament assembly once PMCA amplified using recombinant aSyn. Additionally, aCSF fibril structural assembly resembles the electron density pocket of aggregates found in multiple system atrophy (MSA) patients^[^ ^54^ ^]^ or its atypical form containing Lewi‐MSA hybrid aggregates.^[^ ^51^ ^]^ Although we show symmetrical fibril core that differs from solved ex vivo MSA type 1 and type 2 aggregates that have asymmetrical conformations, the electron density pocket in all mentioned cases is co‐ordinated with identical AA positions (K43, K45, H50).^[^ ^41^, ^54^, ^63^ ^]^ Additionally, there are other cases where lysine and histidine are shown to coordinate the ligand at the side of the aggregate^[^ ^42^ ^]^ or AAs tend to interact with each other within the same protofilament (inter‐rung connection between H50 and K45).^[^ ^64^ ^]^ While it is expected that molecules coordinated with these amino acids may impact the aggregate formation pathway, the appearance of PB Type 2 structure that is closely similar to aCSF Type 1 implies that the coordinated molecules may not have a distinct role in the filament assembly, but instead associate with the pocket after or during its formation. In our case, the probable molecules that may fit the pocket are glucose, glutamine, and phosphate or metal ion complexes. However, more research needs to be done in order to determine the target substrates and understand the impact of pocket‐coordinating molecules to the formation of fibril structures, especially when the instability of filaments complicates their extraction from the solution, thus limiting the likelihood of determining the ligand.

Another important matter to consider is the aSyn aggregate effect on neuroblastoma cells. aCSF fibrils tended to adhere to the cell surface significantly more than those of a control group (PB). There is evidence that specific ions, such as calcium or zinc, found in cerebrospinal fluid, alter aSyn fibril morphology and promote different aggregation pathways. These fibrils exhibit adhesive properties to cell membrane and internal structures, such as pre‐synaptic vesicles,^[^ ^32^, ^58^, ^65^ ^]^ where Me^2+^ concentration fluctuation may occur.^[^ ^66^, ^67^ ^]^ In fact, our Cryo‐EM data show that the outer surface of the fibrils carries positively charged areas, which can be attracted to a negatively charged cell membrane.^[^ ^68^ ^]^ Moreover, structural features such as hydrophobic pockets and β‐sheet stacking could influence their interaction with cell membranes,^[^ ^39^, ^56^ ^]^ enhancing affinity to the lipid bilayer or cellular components. This may lead to extensive cellular internalization, membrane disruption, or oxidative stress.^[^ ^69^, ^70^, ^71^ ^]^ Additionally, the cell toxicity assay revealed an interesting tendency. While cell metabolic activity was reduced at higher aggregate concentrations, the LDH release, which mostly describes damage to the cell wall, was significantly higher when a lower aggregate concentration was added. It could be that aSyn fibrils tend to dissociate to smaller fragments at lower concentrations due to excess amounts of cell media components or being prone to mechanistic stress (cell movement, pipetting).^[^ ^72^, ^73^ ^]^ Separated fibrils, albeit their strong affinity toward each other, have less probability to interact (to clump together), resulting in binding directly to the cell membrane and damaging it as smaller, more toxic fragments.^[^ ^74^ ^]^ While LDH release is directly associated with damage of the cell membrane, the MTT assay shows the mitochondria activity, which may be influenced not only by the damaged cells, but also by other factors, including stress.^[^ ^75^, ^76^, ^77^ ^]^ In our case, large clumps of aggregates may cover portions of the cell surface, disrupting cell metabolism, activating surface stress receptors, or even high expenditures of ATP during aggregate internalization.^[^ ^76^, ^78^, ^79^ ^]^ Fibrils bound on the membrane surface may enter the cell via endocytosis or phagocytosis and disrupt the cell homeostasis, induce ROS production, and imbalance of Ca^2+^ that may directly propagate the reduction of cell metabolic activity measured by MTT assay.^[^ ^76^ ^]^ In fact, this directly translates to a more prominent reduction of assay response in the presence of aCSF fibrils that had a higher affinity to the cell membrane.

These findings provide evidence that the molecular and ionic interfaces of cerebrospinal fluid play an important role in fibril morphology, suggesting that fibril structure is environment‐dependent rather than dictated by properties of aSyn secondary structure. Recreating physiological conditions for aggregate extraction and templating may be the key factor enabling our further understanding about neurodegenerative diseases. Furthermore, the ability to seed identical aggregate structures could contribute to creating kits for the detection of disease‐specific biomarkers that are currently are not efficient enough.^[^ ^80^ ^]^

## 4 Conclusion

In conclusion, artificial cerebrospinal fluid has shifted aSyn aggregation toward an environment‐specific conformation that was solved using Cryo‐EM (2.9 Å resolution). Similar fibril filament assembly, including the protofilament secondary structure, was previously observed in samples from patients (6b & 6c), signifying the importance of using physiologically‐relevant fluids in aSyn aggregation studies. The main contributors to this effect were found to be calcium, magnesium ions, and human serum albumin. The latter proved to be the main factor in stabilizing the fibril conformation within the aCSF environment. Moreover, the aCSF fibrils exhibited increased affinity to SH‐SY5Y neuroblastoma cells.

## 5 Experimental Section

### Preparation of aCSF

The preparation of artificial cerebrospinal fluid (aCSF) was described previously.^[^ ^81^ ^]^ The resulting composition of aCSF contains the main components that may have an effect on the protein aggregation process. The compounds containing Na^+^ and K^+^ ions were dissolved as one stock solution called phosphate buffer (further referred as PB (A component)). Meanwhile, the rest of the components were dissolved separately (B‐I) as described (Table S2, Supporting Information). HCl was added to the final 1x aCSF solution to yield a pH of 7.33.

### Expression and Purification of aSyn

Recombinant aSyn was expressed and purified as described in previous work.^[^ ^82^ ^]^ In short, *E.coli* BL(21) Star (DE3) as host cells were transformed with the pDS5 plasmid containing the SNCA gene. Prepared cells were grown overnight at 37 °C, in ZYM‐5052 medium supplemented with 100 µg mL^−1^ ampicillin. Harvested cells were pelleted, resuspended in lysis buffer, and homogenized. Later, the collected supernatant was heat treated at 80 °C for 20 min in a water bath. The resulting suspension was centrifugated. Ammonium sulphate was added to the collected supernatant (to a saturation of 42%) to precipitate the target protein. After centrifugation, the pellet was dissolved and dialyzed. The final solution containing aSyn and 20 mm Tris‐HCl (pH 8.0) (supplemented with 0.5 mm DTT and 1 mm EDTA) buffer solution was loaded onto DEAE sepharose sorbent for ion exchange chromatography. The target protein was eluted by using 20 mm Tris‐HCl (pH 8.0), 1 M NaCl, 0.5 mm DTT, 1 mm EDTA buffer solution. Collected fractions were combined, concentrated, and loaded onto prepacked Hi‐Load 26/600 Superdex 75 pg. size exclusion column that was equilibrated in PB (127 mm NaCl, 1.8 mm KCl, 7.81 mm Na_2_HPO_4_, 3.19 mm NaH_2_PO_4_, 1.2 mm KH_2_PO_4_, pH 7.33). aSyn was eluted at 130 – 155 mL, collected, and adjusted with elution buffer to a final 100 µm concentration. aSyn solution was distributed into tubes (1.0 mL volume) and stored at −20 °C. The prepared aSyn stock solution aliquots were used a single time after thawing, to avoid any potential oligomerization process during re‐freezing conditions.

### Aggregation Experiments

The de novo aggregation of aSyn was performed under various conditions as described (Table S3, Supporting Information). Purified 100 µm aSyn in PB (127 mm NaCl, 1.8 mm KCl, 7.81 mm Na_2_HPO_4_, 3.19 mm NaH_2_PO_4_, 1.2 mm KH_2_PO_4_, pH 7.33) was mixed with 10xPB and 20x‐100x B ‐ I stock solutions (aCSF components), 10 mm Thioflavin T (ThT) stock solution, and MilliQ water to yield a final aSyn concentration of 50 and 100 µm ThT in the final reaction mixture. The prepared final solutions were distributed into Greiner non‐binding 96‐well plates with 80 µL well^−1^, and each well containing a 3 mm glass‐bead. Aggregation kinetics were monitored by using Clariostar Plus plate reader with the following parameters: 37 °C, orbital agitation of 600 RPM, excitation and emission wavelengths of 440 and 480 nm, and ThT fluorescence measured every 5 min. The aSyn aggregation kinetics experiments were conducted for no longer than 30 h.

The seeded aggregation experiments were conducted by preparing a reaction mixture identical to that used for de novo aggregation, with the addition of 10% of an aggregated aSyn sample. No fibril sonication with ultrasound was used to prevent any structural changes due to high temperature and force at the sonication tip. Each individual repeat was distributed across four wells of the 96‐well plate. Seeded aggregation of aSyn was conducted for 19 ‐ 21 h and monitored using the same aggregation protocol as for de novo aggregation process. For Cryo‐EM measurements, preformed aSyn fibrils after de novo aggregation were seeded without adding ThT, in order to prevent the dye interference. For seeded aggregation of where de novo prepared aCSF fibrils were used to initiate the aggregation of aSyn in aCSF and PB solutions, the preformed fibrils were sonicated for 5 min using Bandelin Sonopuls ultrasonic homogenizer with an MS72 tip (20% power, with 10 s sonication / 10 s rest intervals), and the aggregation process was monitored without agitation. The resulting aggregate solutions were placed in non‐binding tubes (Axygen, maximum recovery, ref. MCT‐200‐L‐C) and centrifuged for 10 min at 15 000 x g. The supernatant was used for measuring the unaggregated (soluble) fraction of aSyn in each sample.

### Aggregation End‐Point Measurements

After aggregation was completed, each sample was moved to Greiner non‐binding half area 96‐well plates in order to avoid any interference with the 3 mm glass beads present after the aggregation. Once cooled down to room temperature, the plate without any cover was inserted into the Clariostar Plus plate, and optical density was recorded at 600 nm three times.

Each resulting sample excitation‐emission matrices (EEM) were scanned similarly as described previously.^[^ ^43^ ^]^ In short, emission (470 – 490 nm, 1 nm step) was recorded using 450 nm excitation wavelength, and excitation (440 – 460 nm, 1 nm step) was recorded using 480 nm emission wavelength. The collected data were combined into 3D maps that were called EEM using ClarioStar MARS software. “Center of mass” and the ThT fluorescence intensity values at maximum excitation and emission positions were extracted for each sample and its technical repeats.

### Fibril Restructurization Measurements

The aCSF aSyn sample was centrifugated for 10 min at 14 000 x g, after which 90 % of the supernatant volume was removed. The concentrated fibril solution was placed in Corning non‐binding 96‐well plates (15 µL solution and one 3 mm glass bead in each well). The wells were then supplemented with either 135 µL aCSF or PB solutions (each containing 50 µm ThT), followed by brief pipetting. Immediately after the mixing procedure, the sample EEM were scanned as mentioned in the aggregation end‐point measurements section. The scanning procedure was repeated every 60 s for 30 min with 5 s 600 RPM agitation before each measurement. EEM intensity maximum positions and values were determined as described previously.^[^ ^43^ ^]^

### Aggregate Resuspension in Buffer A and Different aCSF Composition

100 µL of the final aggregate solution was placed in 250 µL PCR tubes that were centrifugated 5 min at 9000 RPM (ThermoFisher Mini Centrifuge). Then, the supernatant was removed and the fibrils were resuspended with 100 µL of Buffer A or a different aCSF composition solution. This process was repeated once again before imaging of the sample was conducted.

### Fourier‐Transform Infrared Spectroscopy (FTIR)

The aSyn fibril solutions were centrifuged for 10 min at 15 000 x g. The supernatant was removed, and the remaining fibril pellets were resuspended in 200 µL of D2O (containing 300 mm NaCl).^[^ ^83^ ^]^ The centrifugation and resuspension into D2O were repeated three times. After the final centrifugation, the supernatant was removed, and the remaining sample was resuspended in 80 µL of D2O with 300 mm NaCl. The measurements were conducted as described previously.^[^ ^43^ ^]^ All data processing was performed using QUASAR software.^[^ ^84^ ^]^

### Atomic Force Microscopy (AFM)

aSyn fibrils formed in Buffer A and aCSF were used to prepare samples for AFM imaging, similarly as previously described.^[^ ^81^ ^]^ In short, freshly cleaved mica was functionalized by placing 40 µL of 0.5 % (v/v) APTES (Sigma‐Aldrich, cat. No. 440 140) in MilliQ water and incubating for 5 min. Then the mica was washed with 2 mL MilliQ water and dried under gentle airflow. 40 µL of each diluted sample (diluted to 20 µm aSyn concentration) was added to individual mica, incubated for 5 min, washed, and dried as previously described. The AFM imaging was done using a Dimension Icon (Brucker) atomic force microscope. Images of 1024 x 1024 pixel resolution were recorded using Nanoscope 10.0 (Bruker) operating in tapping mode and equipped with a silicon tip (Tap300AI‐G; 40 Nm^−1^, Budget Sensors). Image levelling, corrections, cropping, and analysis were done using Gwyddion 2.63 software.^[^ ^85^ ^]^ The fibril profile heatmaps were drawn using Origin 2018. Gaussian fitting was done in order to approximate fibril height and width traces, which were used to calculate Max‐Min and Pitch parameters.

### High‐Speed Atomic Force Microscopy (HS‐AFM)

aSyn fibrils formed in aCSF were placed on the APTES‐functionalized mica and incubated for 5 min. Then the mica was washed with aCSF solution and was placed in a custom measurement cell (100 µL) with aCSF or PB solutions. The imaging was done using HS‐AFM (SS‐NEX, RIBM, Japan). Images of 250 x 250 pixel resolution were recorded using IgorPro‐based RIBM software (Ibis v.1.0.2.4, IgorPro v.6.3.7.2). The system operated in tapping mode and was equipped with ultrashort (8 µm) silicon nitride (Si_3_N_4_) rectangular cantilevers (BL‐AC10DS‐A2, Olympus, Japan) with a tip radius of 24 nm. These cantilevers had a nominal spring constant of ≈0.1 N m^−1^ and a resonant frequency of ≈0.5 MHz in solution. Data analysis was performed using NanoLocz (v.1.20) software.^[^ ^86^ ^]^

### Cryo‐Electron Microscopy (Cryo‐EM) data collection

For Cryo‐EM sample preparation, 3 µL of alpha synuclein fibrils were applied to the glow‐discharged holey carbon Cu grids (Quantifoil) and blotted with filter paper using Vitrobot Mark IV (FEI Company). The grids were immediately plunge‐frozen in liquid ethane and clipped. Cryo‐EM data were collected either on a Glacios transmission electron microscope (Fisher Scientific) operated at 200 kV camera or a Krios transmission electron microscope (Fisher Scientific) operated at 300 kV and equipped with Falcon 3EC/4i cameras. Cryo‐EM data collection statistics can be found in Table S4 (Supporting Information).

### Image Pre‐Processing and Helical Reconstruction

The micrographs were aligned, motion corrected using MotionCorr2 1.2.1^[^ ^87^ ^],^ and the contrast transfer function was estimated by CTFFIND4.^[^ ^88^ ^]^ The fibrils were picked, and all subsequent 2D classifications were performed in Relion 5.0.^[^ ^89^ ^]^ Distribution of polymorphs was identified by FilamentTools (https://github.com/dbli2000/FilamentTools), which was primarily referred in Tau^[^ ^90^, ^91^ ^]^ structural studies. Filament tools are integrated as a part of the Relion software. It uses UPGMA (Unweighted Pair Group Method using arithmetic Averages) hierarchical clustering to sort particles, which were assigned to different filament numbers. The output includes the number of filaments for each cluster, which were used to plot pie charts. The sensitivity of clustering was controlled by two paraments (threshold and minimum number of particles for a cluster) that were manually adjusted until a good separation was observed. The analysis dendrograms are presented in Figures S6–S9 (Supporting Information). Segments with a box size of 1024 downscaled to 384 were used to generate de novo model using the relion_helix_inimodel2d function. Next, 3D auto‐refinements were performed with optimisation of the helical twist and rise parameters once the resolutions extended beyond 4.7 Å. To improve the resolution, Bayesian polishing and CTF refinement were performed. Final maps were sharpened using standard post‐processing procedures in RELION (Figure S20, Supporting Information). Helical model parameters can be found in Table S4 (Supporting Information).

### Determination of Fibril FWHM

The initial full width at half maximum (FWHM) of fibrils was calculated from intensity profile plots of 2D class projections. Profile data were extracted in Fiji and analyzed by fitting Gaussian functions to either single or double peaks, depending on the profile shape.

### Model Building and Refinement

The atomic model was built de novo in Coot^[^ ^92^ ^]^ and was subjected to several real‐time refinements in PHENIX^[^ ^93^ ^]^ with manual curation of outlines to ensure energy‐favored geometry. Map volume and models were visualized using the Chimerax program.^[^ ^94^ ^]^ Hydrophilicity and hydrophobicity of the model were inspected with the ProCart web server.^[^ ^95^ ^]^

### Cell Culturing

Cell culture used for experiments (SH‐SY5Y human neuroblastoma cells) was obtained from the American Type Culture Collection (ATCC, Manassas, VA, USA). The cells were grown in Dulbecco's Modified Eagle Medium (DMEM) (Gibco), supplemented with 10% Fetal Bovine Serum (FBS) (Sigma‐Aldrich), 1% Penicillin–Streptomycin (10 000 U mL^−1^) (Gibco) at 37 °C in a humidified, 5% CO_2_ atmosphere in a CO_2_ incubator.

### MTT and LDH Assay

The samples for cell assays were collected from seeded aggregation experiments. To avoid any possible effect of reaction buffers (PB and aCSF), samples containing aSyn fibrils were centrifuged for 1 h at 15 000 x g. The supernatant was removed, and the remaining fibril pellet was resuspended in the same volume of DMEM medium (for MTT assay) or in Advanced DMEM (for LDH assay).

For both cell assays, SH‐SY5Y cells were seeded in a 96‐well plate (≈15 000 cells well^−1^) and incubated overnight. In case of the MTT assay, after the incubation cell medium was changed to one containing aSyn fibrils formed either in PB or in aCSF. For each of the conditions titration of sample was applied to yield the final concentrations of 20, 10, 5, and 1 µM. After 48 h of incubation 10 µm of 3‐(4,5‐dimethylthiazol‐ 2‐yl)‐2,5‐diphenyltet‐tetrazolium bromide (MTT) reagent (12.1 mm in PBS) was added to each well and followed by 2 h of incubation. To dissolve formazan crystals 100 µL of 10% SDS with 0.01 N HCl solution was added to each well. After 2 h, the absorbance was measured at 570 and 690 nm (as reference wavelength) using a Clariostar Plus plate reader.

Meanwhile, LDH release into the medium was quantified by using the Cytotoxicity Detection kit (Roche). After overnight incubation, the medium inside the wells was aspirated, and 100 µL well^−1^ of Advanced DMEM (Gibco) was added. Then, each sample was diluted in Advanced DMEM. Prepared samples were added to the wells to reach 200 µL well^−1^ of total medium volume, which resulted in the final 20, 10, 5, and 1 µM, sample concentrations for each condition. After 24 h of incubation, 100 µL of the medium from each well was aspirated, centrifugated, and transferred into a flat bottom 96‐well test plate. Freshly prepared LDH reagent was added and incubated for 30 min at room temperature. The absorbance was measured at 492 nm and 600 nm (as reference wavelength) using a Clariostar Plus plate reader.

### Cell Imaging

The aSyn fibrils for cell imaging were pre‐formed by using mCherry‐aSyn fluorescent protein. Here, the seeded aggregation experiment was conducted as described in the Experimental Section Aggregation Experiments, with the addition of 5% mCherry‐aSyn.

The SH‐SY5Y cells were seeded in a 24‐well plate (≈90 000 cells well^−1^) and incubated overnight. Samples containing mCherry‐aSyn in PB and mCherry‐aSyn in aCSF were centrifuged for 1 h at 15 000 x g. Pelleted fibrils of each sample were resuspended in FluoroBrite DMEM (Gibco) and further diluted to a final concentration of 20 or 1 µM. After the overnight incubation with samples, cells were analysed with EVOS FL Auto fluorescence microscope (Life Technologies, USA) by taking photos with 20x and 40x objectives. Here, two types of data were collected: “before” and “after”. For “after” imaging, media containing aSyn samples was removed, cells washed with phosphate‐buffered saline (PBS, Gibco), and replaced with new FluoroBrite media.

All images were analyzed using the Fiji package for ImageJ.

### Statistical Analysis

The de novo aSyn aggregation kinetic data (lag time and apparent rate) were analyzed by fitting the kinetic curves using Boltzmann's sigmoidal equation.^[^ ^43^ ^]^ The seeded aggregation kinetic curves were normalized and then analyzed by linear fit. Halftime and slope parameters were extracted from the fitted data. Each condition was repeated 8 times (technical repeats) to evaluate the reproducibility of the data.

Each SDS‐Page gel was stained using GelCodeTM Blue Safe Protein Stain (Thermo Scientific) and scanned using ChemiDocTM MP Imaging System (Bio‐Rad). The SDS‐PAGE images were imported to GelAnalyzer 23.1.1 software (available at) by Istvan Lazar Jr., PhD, and Istvan Lazar Sr., PhD, CSc. Then, basic operations (cropping, gel rotation) were done to properly align the gel for analysis. The parallel lines with 60% of lane width with adjusted curving were drawn. Each band (peak) was integrated using valley‐to‐valley baseline integration mode, and their corresponding areas were normalized based on the standard HSA concentration present in aCSF solution (buffer solution only). Technical sample repeats were displayed in box plots of 25 – 75 % range, whisker range was 1.5 SD, line within represents median or bar graphs with error bars of one standard deviation.

The statistical analysis of MTT and LDH assay results was conducted by using ORIGIN 2018 software (OriginLab Corporation, MA, USA), One‐way analysis of variance (ANOVA) and Bonferroni means comparison (n = 9) for each condition were conducted to determine differences between sample concentrations (*p < 0.05, **p < 0.01, ***p < 0.001). Three independent sets of samples with three repeats of each condition were used for MTT and LDH assays.

## Conflict of Interest

The authors declare no conflict of interest.

## Author Contributions

D.S. did conceptualization, methodology, validation, formal analysis, investigation, Writing ‐ Original Draft, Writing ‐ Review & Editing, supervision, and funding acquisition. A.S. did conceptualization, methodology, validation, formal analysis, investigation, data curation, Writing ‐ Original Draft, Writing ‐ Review & Editing, visualization, supervision, and project administration. R.S. did methodology, formal analysis, investigation, Writing ‐ Original Draft, Writing ‐ Review & Editing, and visualization. R.T. did methodology, investigation. M.Z. did methodology, validation, formal analysis, investigation, and Writing ‐ Review & Editing. A.J. did an investigation. U.V. did the investigation, Writing, Review, and Editing. V.S. did resources, Writing ‐ Review & Editing, and funding acquisition.

## Supporting information



Supporting Information

Supplemental Video 1

Supplemental Video 2

Supplemental Video 3

Supplemental Video 4

## Data Availability

All data needed to evaluate the conclusions in the paper are present in the paper and/or Supporting Materials. The raw data used in this paper have been tabulated and are available on Mendeley Data: (10.17632/d3cby7cv57/3). The reconstructed cryo‐EM map was deposited in the Electron Microscopy Data Bank with the accession codes EMD‐52833 The coordinates of the fitted atomic model were deposited in the Protein Data Bank under the accession code 9IC7. All other relevant data are available from the corresponding author upon reasonable request.
