# Supplementary material for: Formation of Condition‐Dependent Alpha‐Synuclein Fibril Strain in Artificial Cerebrospinal Fluid
Source: Adv Sci (Weinh). 2025 Nov 20;13(7):e05228. doi: 10.1002/advs.202505228 (PMC12866731; doi:10.1002/advs.202505228)
Supplement: Supplementary file 1 — Supporting Information [file ADVS-13-e05228-s001.docx]

**Formation of Condition-Dependent Alpha-Synuclein Fibril Strain in Artificial Cerebrospinal Fluid**

Rūta Sniečkutė^1^, Darius Šulskis^1^, Arūnė Jocytė^1^, Urtė Venclovaitė^1^, Rimgailė Tamulytė^2^, Mantas Žiaunys^1^, Vytautas Smirnovas^1^, Andrius Sakalauskas^1^*

^1^Institute of Biotechnology, Life Sciences Center, Vilnius University, Vilnius, Lithuania

^2^Institute of Biochemistry, Life Sciences Center, Vilnius University, Vilnius, Lithuania

*To whom correspondence should be addressed:
Andrius Sakalauskas
[Andrius.sakalauskas@gmc.vu.lt](mailto:Andrius.sakalauskas@gmc.vu.lt)

Table 1. ANOVA means comparison of aSyn aggregates effect to cell. The comparison is done between equal concentration of fibrils that were produced at different conditions (aCSF and PB). “NS” corresponds to not significant.

|  | *Significance* | |
| --- | --- | --- |
| **aSyn concentration (µM)** | **MTT** | **LDH** |
| 20 | 0.001 | NS |
| 10 | 0.001 | NS |
| 5 | 0.001 | NS |
| 1 | 0.001 | NS |

Table 2. Composition of aCSF.

|  | **Concentration in 10x solution, mM** | | | | | | | | | **1x, mM** |
| --- | --- | --- | --- | --- | --- | --- | --- | --- | --- | --- |
| ***Component*** | **PB (A)** | ***B*** | ***C*** | ***D*** | ***E*** | ***F*** | ***G*** | ***H*** | ***I*** | **aCSF** |
| NaCl | 1270 |  |  |  |  |  |  |  |  | 127 |
| KCl | 18 |  |  |  |  |  |  |  |  | 1.8 |
| Na_2_HPO_4_ | 78.1 |  |  |  |  |  |  |  |  | 7.81 |
| NaH_2_PO_4_ | 31.9 |  |  |  |  |  |  |  |  | 3.19 |
| KH_2_PO_4_ | 12 |  |  |  |  |  |  |  |  | 1.2 |
| Urea |  | 6.5 |  |  |  |  |  |  |  | 0.65 |
| L-glutamine |  |  | 7 |  |  |  |  |  |  | 0.7 |
| HSA |  |  |  | 0.0615 |  |  |  |  |  | 0.00615 |
| Cholesterol |  |  |  |  | 0.052 |  |  |  |  | 0.0052 |
| Sodium lactate |  |  |  |  |  | 24 |  |  |  | 2.4 |
| CaCl_2_ |  |  |  |  |  |  | 14 |  |  | 1.4 |
| MgCl_2_ |  |  |  |  |  |  |  | 13 |  | 1.3 |
| Glucose |  |  |  |  |  |  |  |  | 40 | 4 |

Table 3. Experimental design of aggregation study. The grey box marks the missing components from the final reaction mixture.

|  | | **PB (A)** | ***B*** | ***C*** | ***D*** | ***E*** | ***F*** | ***G*** | ***H*** | ***I*** | ***aSyn*** | ***ThT*** |
| --- | --- | --- | --- | --- | --- | --- | --- | --- | --- | --- | --- | --- |
| Reaction mixtures | aCSF |  |  |  |  |  |  |  |  |  |  |  |
|  | aCSF - urea |  |  |  |  |  |  |  |  |  |  |  |
|  | aCSF - glutamine |  |  |  |  |  |  |  |  |  |  |  |
|  | aCSF - HSA |  |  |  |  |  |  |  |  |  |  |  |
|  | aCSF - cholesterol |  |  |  |  |  |  |  |  |  |  |  |
|  | aCSF - sodium lactate |  |  |  |  |  |  |  |  |  |  |  |
|  | aCSF – CaCl_2_ |  |  |  |  |  |  |  |  |  |  |  |
|  | aCSF – MgCl_2_ |  |  |  |  |  |  |  |  |  |  |  |
|  | aCSF - glucose |  |  |  |  |  |  |  |  |  |  |  |
|  | ***PB*** |  |  |  |  |  |  |  |  |  |  |  |
|  | ***PB*** + glucose |  |  |  |  |  |  |  |  |  |  |  |
|  | ***PB*** + MgCl_2_ |  |  |  |  |  |  |  |  |  |  |  |
|  | ***PB*** + CaCl_2_ |  |  |  |  |  |  |  |  |  |  |  |
|  | ***PB*** + sodium lactate |  |  |  |  |  |  |  |  |  |  |  |
|  | ***PB*** + cholesterol |  |  |  |  |  |  |  |  |  |  |  |
|  | ***PB*** + HSA |  |  |  |  |  |  |  |  |  |  |  |
|  | ***PB*** + glutamine |  |  |  |  |  |  |  |  |  |  |  |
|  | ***PB*** + urea |  |  |  |  |  |  |  |  |  |  |  |

Table 4. Cryo-EM data collection and modelling statistics.

|  | Alpha-synuclein in aCSF | Alpha-Synuclein in PBS, Type 1/2/3 | Asyn_ascf_ seeded to PB conditions | Alpha-synuclein in aCSF (repeat) |
| --- | --- | --- | --- | --- |
| **Data collection** |  |  |  |  |
| Magnification | 165000 | 150000 | 92000 | 150000 |
| Pixel size (Å) | 0.754 | 0.95 | 1.1 | 0.95 |
| Defocus range (μm) | -2.0 to -1.0 | -2.2 to -1.2 | -2.2 to -1.2 | -2.2 to -1.2 |
| Voltage (kV) | 300 | 200 | 200 | 200 |
| Camera | Falcon 4i | Falcon 4i | Falcon 3CE | Falcon 4i |
| Microscope | Krios | Glacios 2 | Glacios | Glacios 2 |
| Energy filter slit width (eV) | 10 | - | - | - |
| Exposure time | 3.37 | 3.99 | 46.33 | 3.99 |
| Number of eer fractions | 30 | 40 | 30 | 40 |
| Total dose (e^-^/Å^2^) | 22.65 | 30 | 30 | 30 |
| **Data processing** |  |  |  |  |
| Micrographs | 3510 | 3199 | 857 | 1433 |
| Picked particles | 161452 | 6516 | 1014 | 468 |
| Box size (pixel) | 384 | 384 or 1024 downscaled to 256 | 1024 downscaled to 256 | 1024 downscaled to 256 |
| Inter-box distance (Å) | 18.9 | 30/20 | 17.27 | 20 |
| Segments extracted | 1253042 | 122335/33022 | 6750 | 2058 |
| Segments used for 3D final (or initial for Alpha-Synuclein in PBS) reconstruction | 20772 | 68016/13480/11952 |  | - |
| Helical twist (º) | -1.11 | -1.22/-1.14/-1.1/ |  | - |
| Helical rise (Å) | 4.91 | 4.75/4.75/4.75 |  | - |
| Symmetry imposed | C2 | C1/C2/C2 |  | - |
| Map resolution FSC 0.143 (Å) | 2.9 |  | - | - |
| **Refinement** |  |  |  |  |
| Model resolution (Å)  FSC threshold  Map sharpening factor (Å2) | 2.9  0.143  -42.2007 | - |  |  |
| **Model**  **composition** |  |  |  |  |
| Non-hydrogen atoms  Protein residues  Ligands | 9688  1386  0 | - | - |  |
| B factors (Å^2^) min/max/mean | 14.10/96.52/48.71 |  | - |  |
| **R.m.s.**  **deviations** |  |  |  |  |
| Bond lengths (Å)  Bond angles (°) | 0.003  0.504 | - | - |  |
| **Validation** |  |  |  |  |
| MolProbity score  Clashscore  Rotamer outliers (%) | 2.23  6.02  4.35 | - | - |  |
| **Ramachandran plot** |  |  |  |  |
| Favored (%)  Allowed (%)  Disallowed (%) | 0  6.19  93.81 | - | - |  |


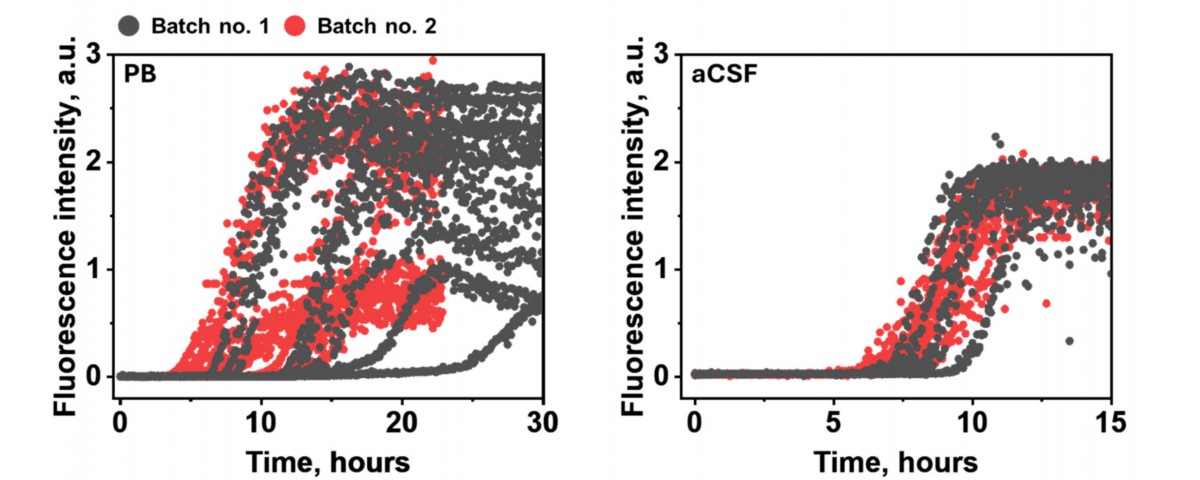


Figure 1. De novo aggregation of aSyn in PB and aCSF using two different aSyn batches. The aggregation reaction mixtures were prepared separately and the reactions conducted independently in order to clarify the batch-to-batch repeatability of the results. Each condition consisted of separate technical repeats (n = 8).


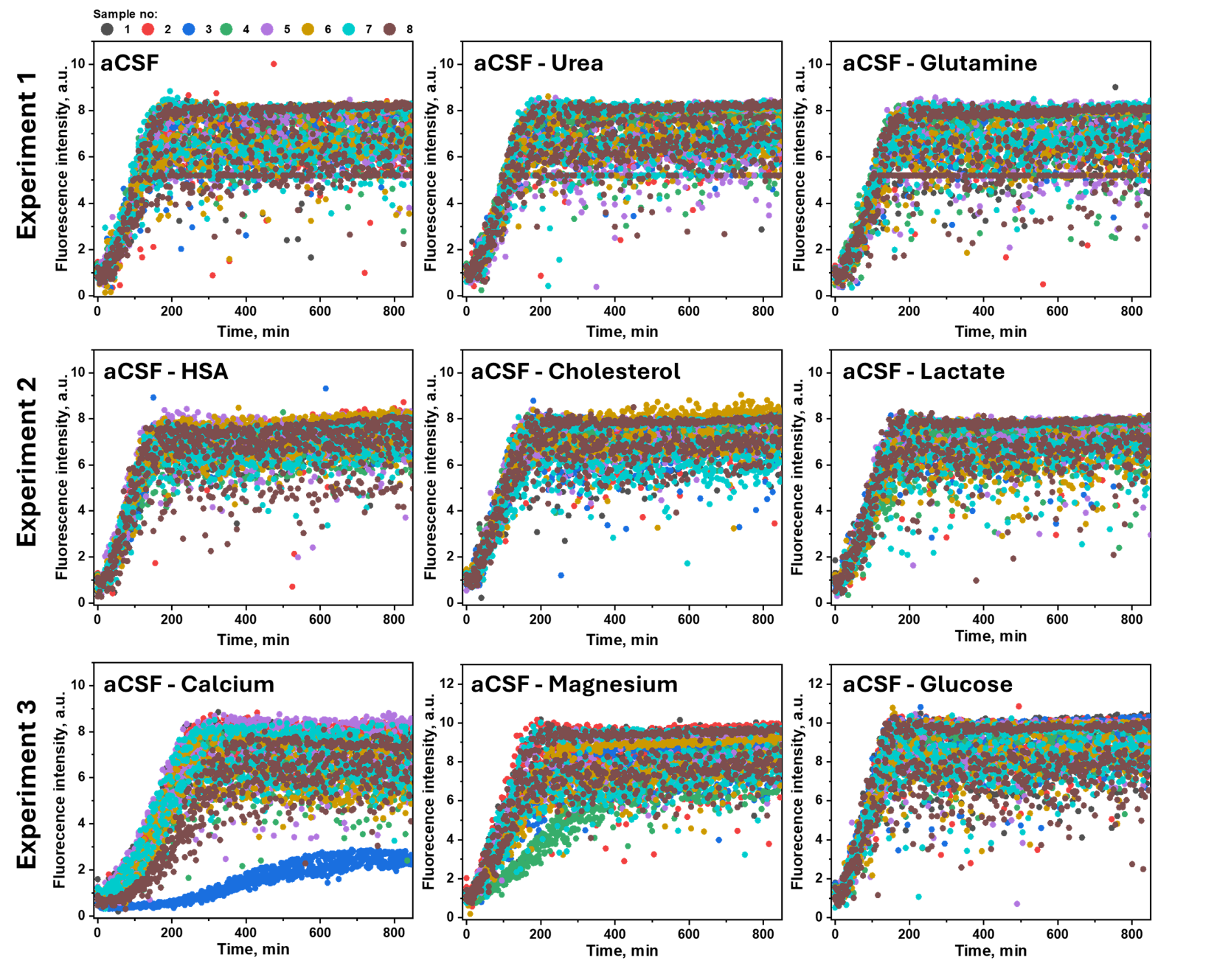


Figure 2. Seeded aggregation kinetics of aSyn. After de novo aggregation, aggregates formed in each condition were used to seed the monomer aSyn (10% of seeds added). For data measurements (experiments 1 - 3), enhanced dynamic range was used, disallowing the fluorescence intensity comparison due to automatic fluorescence intensity adjustments. Each sample (1 – 8) in each condition consisted of four technical repeats.


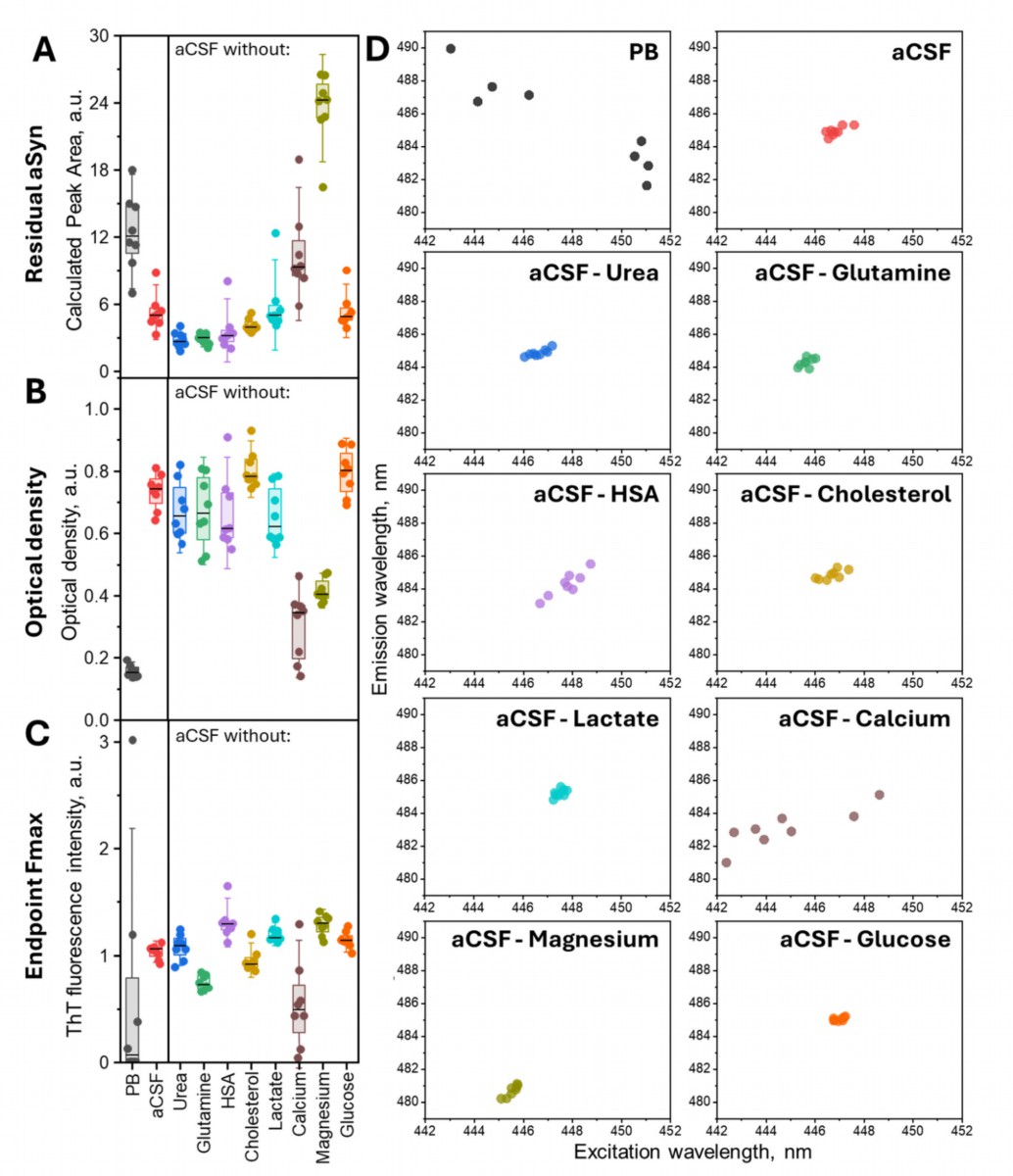


Figure 3. Extracted endpoint concentration of aSyn (A) based on SDS-PAGE images using GelAnalyzer 23.1.1 software. The normalization between different SDS-PAGE images was done according to two standard samples with reference to HSA concentration. The average of three aggregation endpoint optical density measurements (B) for each sample technical repeat is displayed in the box plots. Endpoint maximum fluorescence intensity (C) for each technical repeat at their corresponding excitation – emission maximum positions (D) are displayed in the box plots. Each box plot is of 25 – 75 % range, whisker range is 1.5 SD, line within represents median.


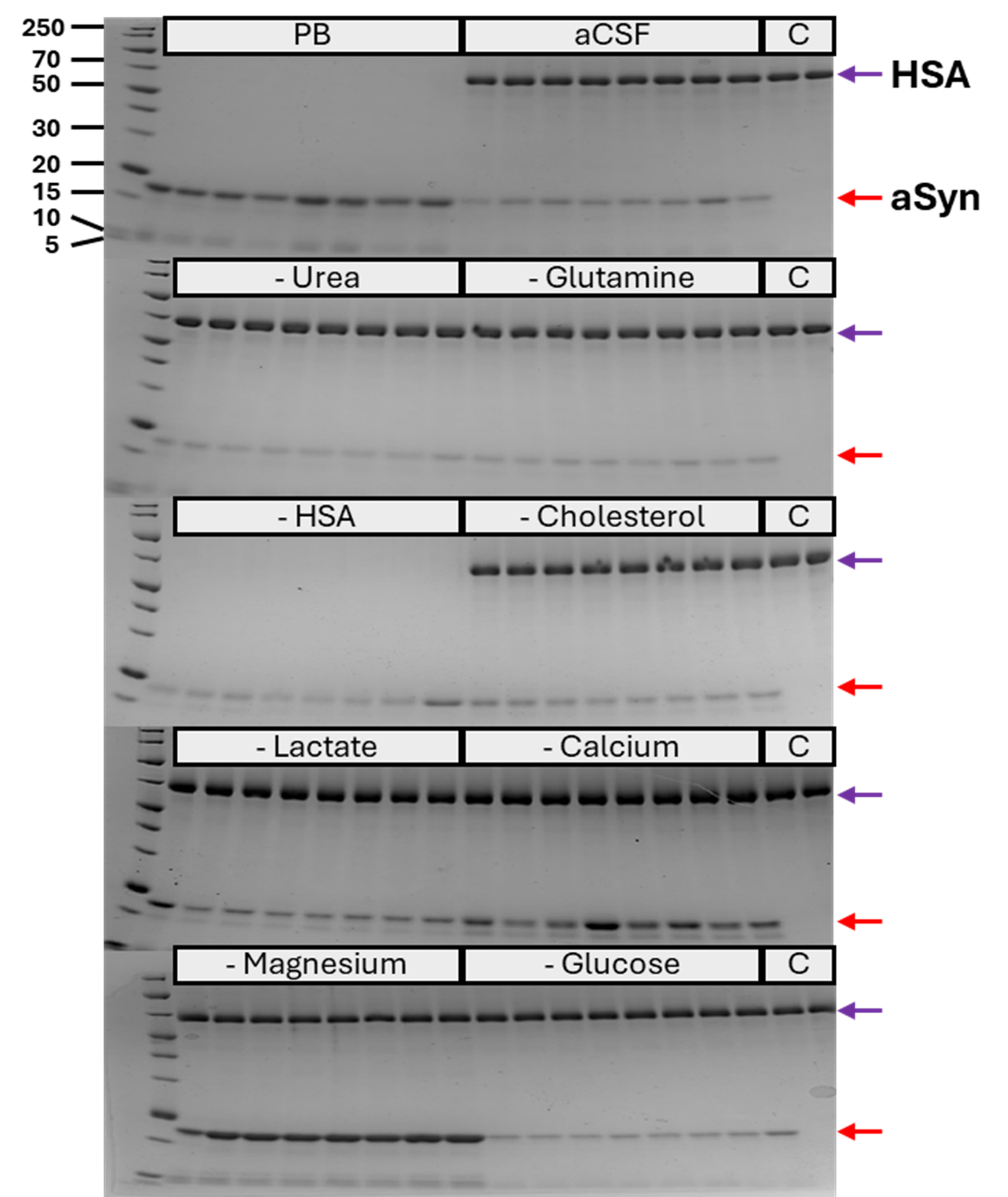


Figure 4. After protein aggregation was completed, resulting fibrils in the mixtures were pelleted and the supernatant was tested on SDS-PAGE. The sample preparation was done as described in the materials and methods section. The molecular weight marker was used (PageRuler^TM^ Unstained Broad Range Protein Ladder) to identify positions of HSA (purple arrow) and aSyn (red arrow). Original SDS-PAGE gels are added to 10.17632/d3cby7cv57/3


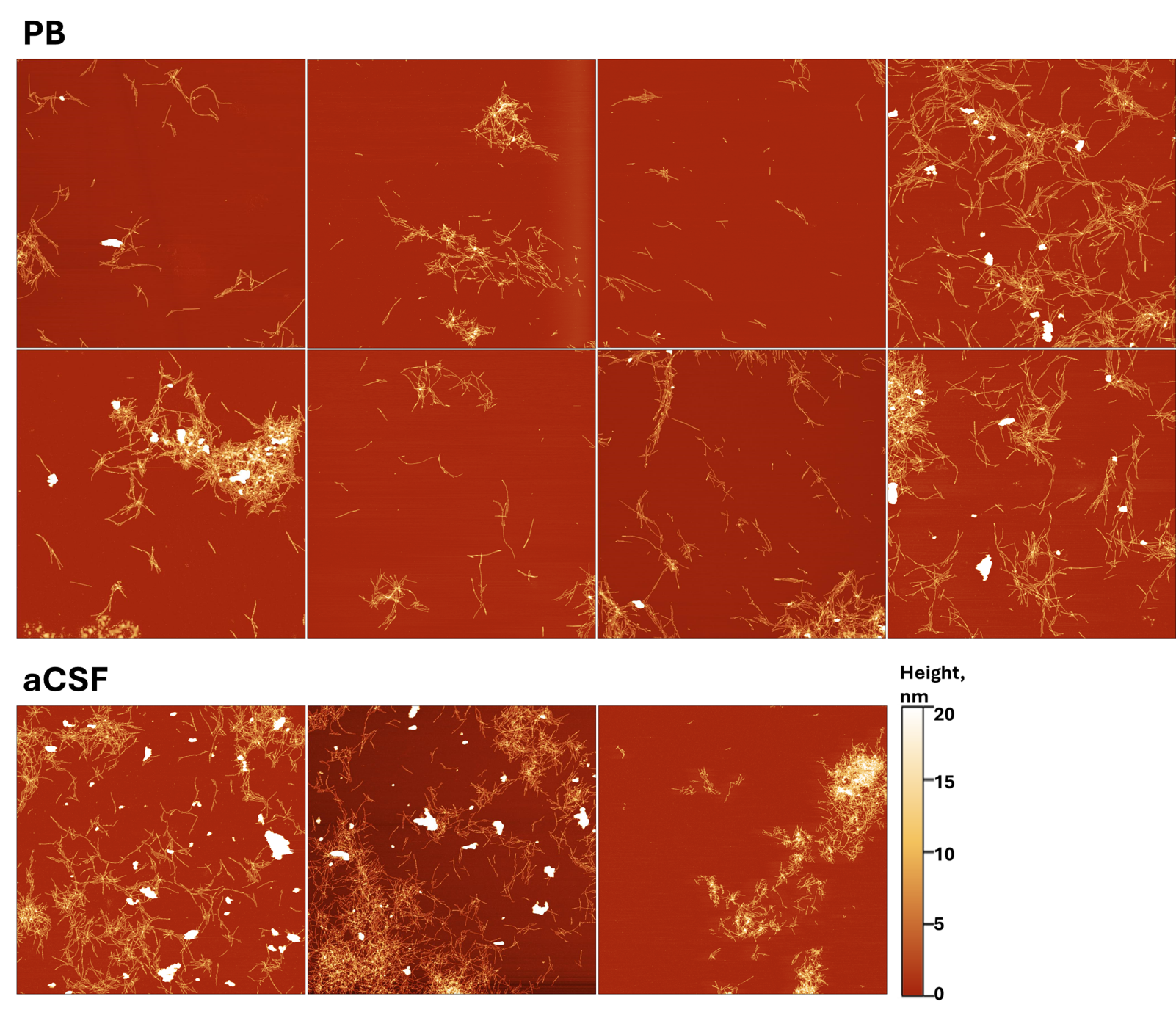


Figure 5. aSyn fibrils made in PB and aCSF conditions. Original micrographs of 10 µM x 10 µM are displayed.


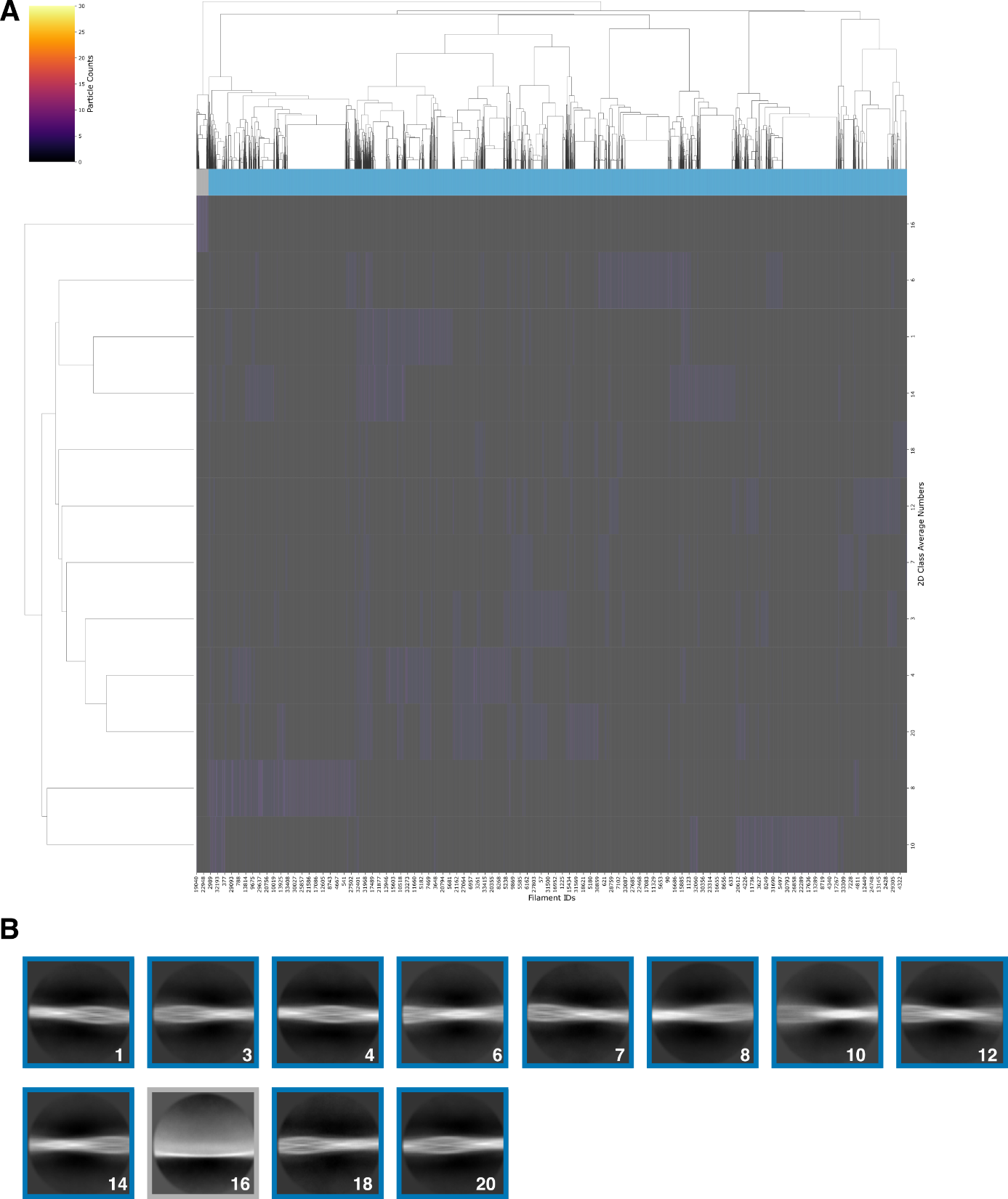


Figure 6. (A) Hierarchical classification of aCSF filament segments according to their assigned 2D class average (vertical) and the picked filament ID (horizontal). (B) 2D classes averages and their corresponding numbers. Data set characteristics are presented in Table 4.


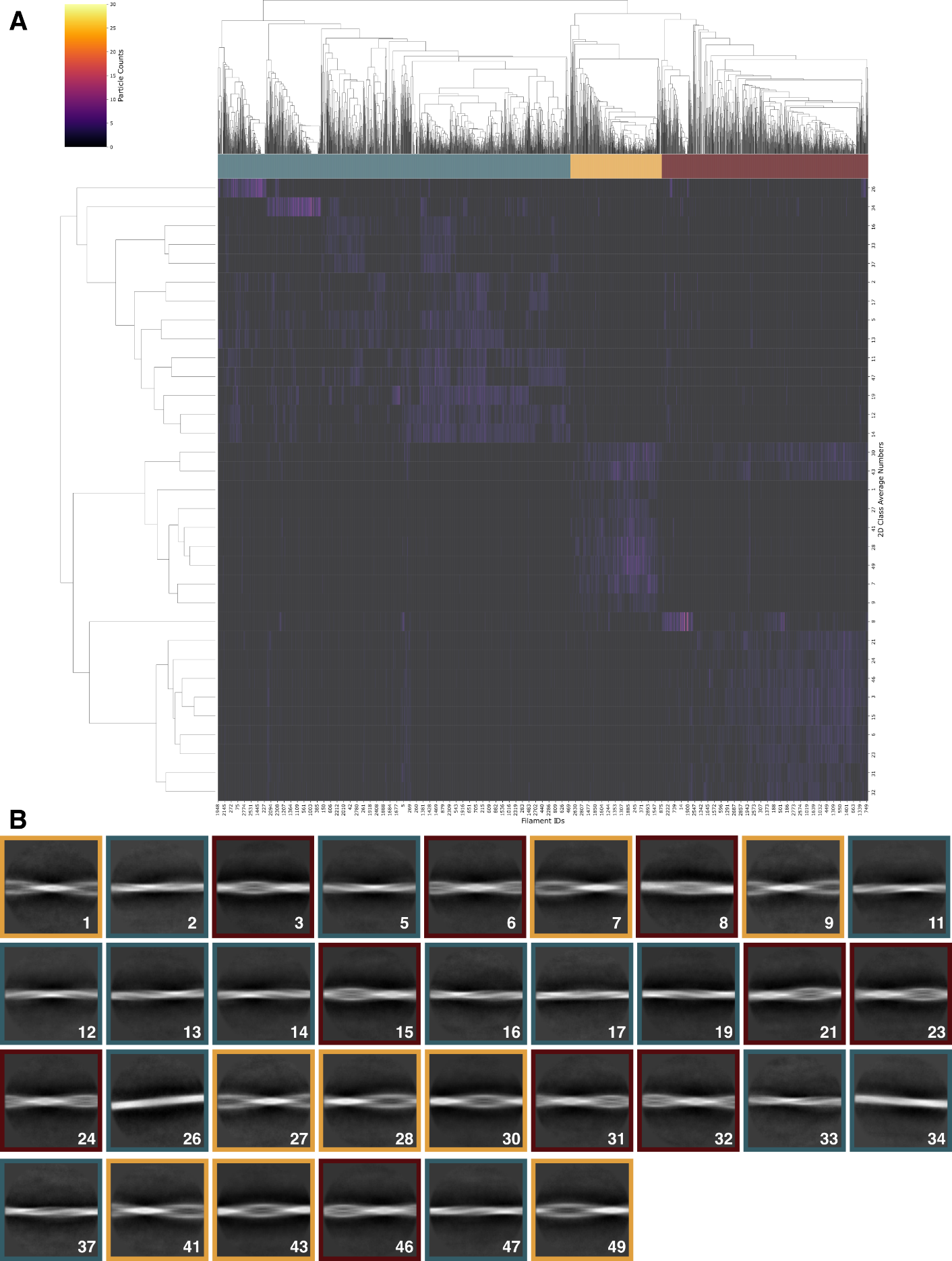


Figure 7. (A) Hierarchical classification of PB filament segments according to their assigned 2D class average (vertical) and the picked filament ID (horizontal). (B) 2D classes averages and their corresponding numbers. Data set characteristics are presented in Table 4.


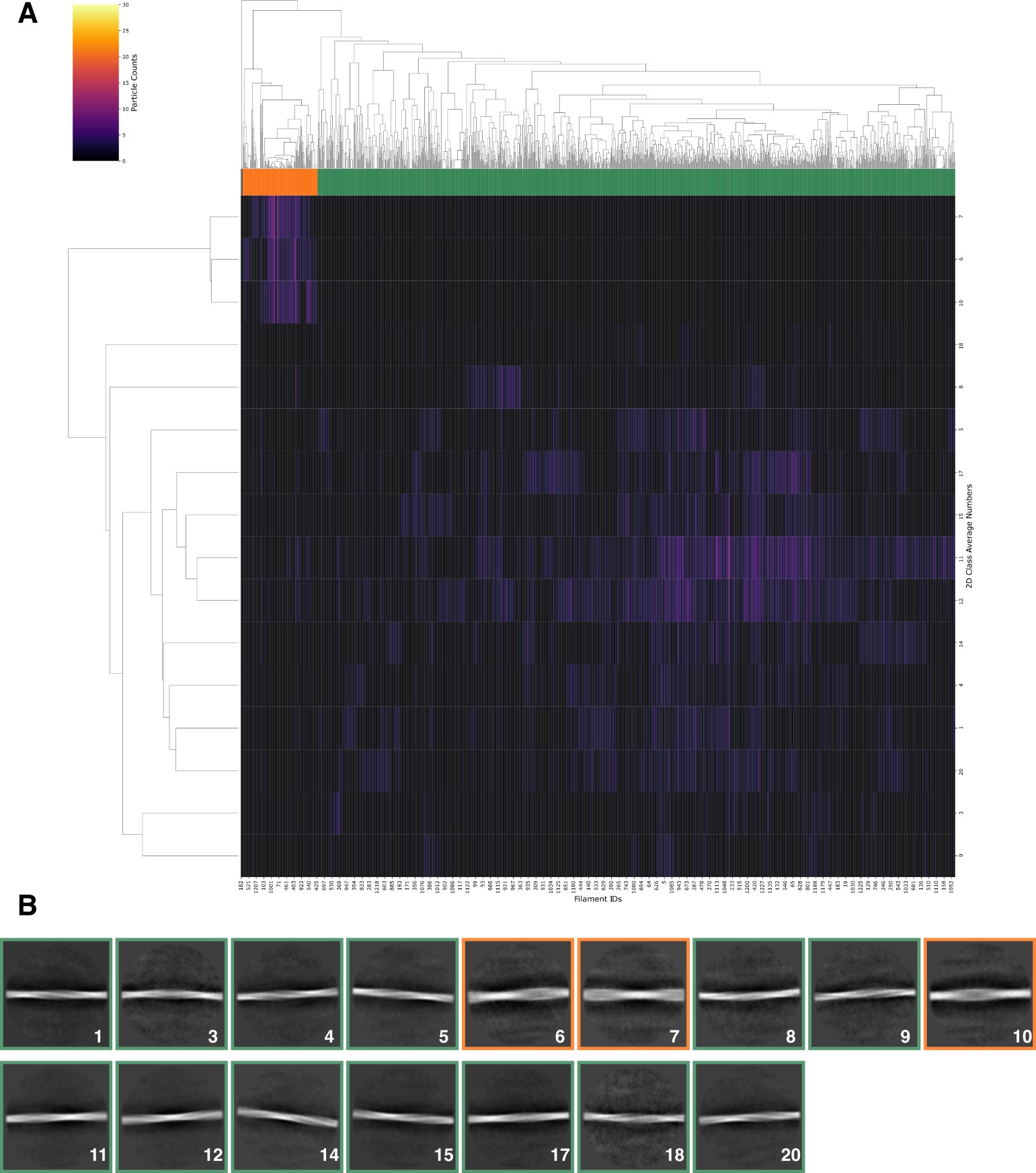


Figure 8. (**A**) Hierarchical classification of aCSF fibrils seeded in PB segments according to their assigned 2D class average (vertical) and the picked filament ID (horizontal). (**B**) 2D classes average and their corresponding numbers. Data set characteristics are presented in Table 4.


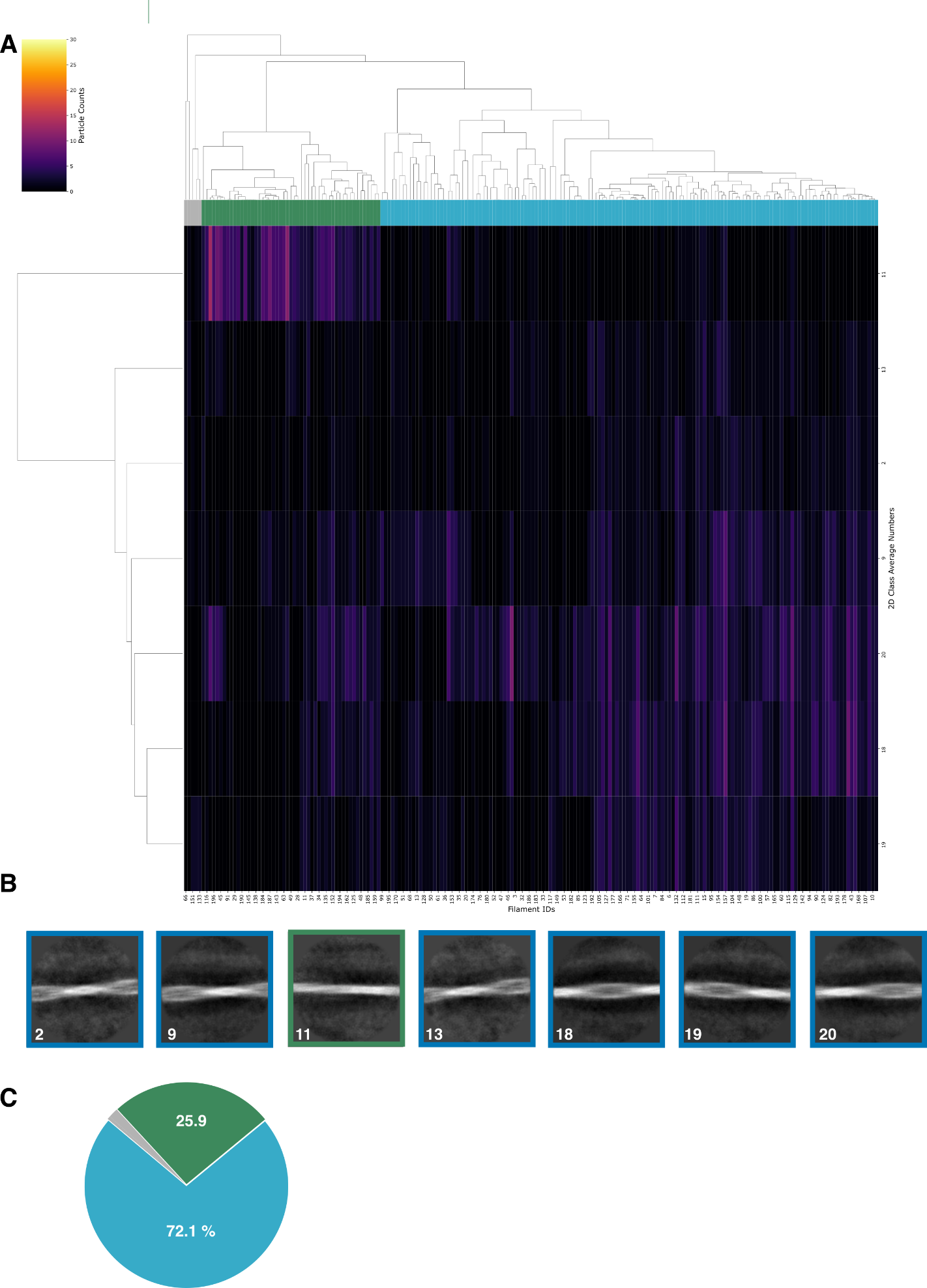


Figure 9. (A) Hierarchical classification of aCSF fibrils (repeated experiment) segments according to their assigned 2D class average (vertical) and the picked filament ID (horizontal). (B) 2D classes average and (C) their corresponding numbers. Data set characteristics are presented in Table 4.


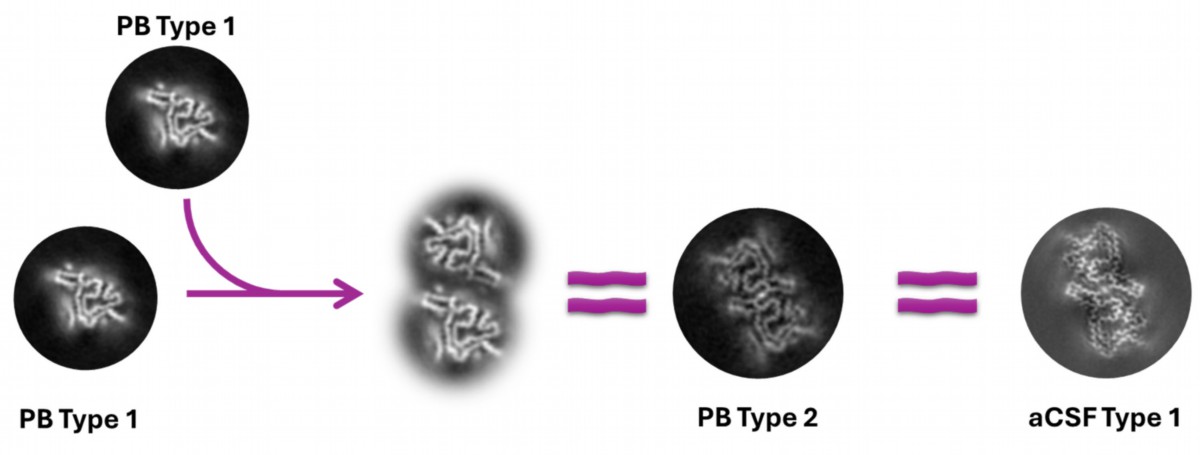


Figure 10. Basic representation of PB Type 1 assembly similarity to PB Type 2 and aCSF Type 1 structures. The expected assembly of PB Type 1 filament results in PB Type 2 aggregates that are seemingly similar to structures found in aCSF. The cross-section maps do not represent equal scaling.


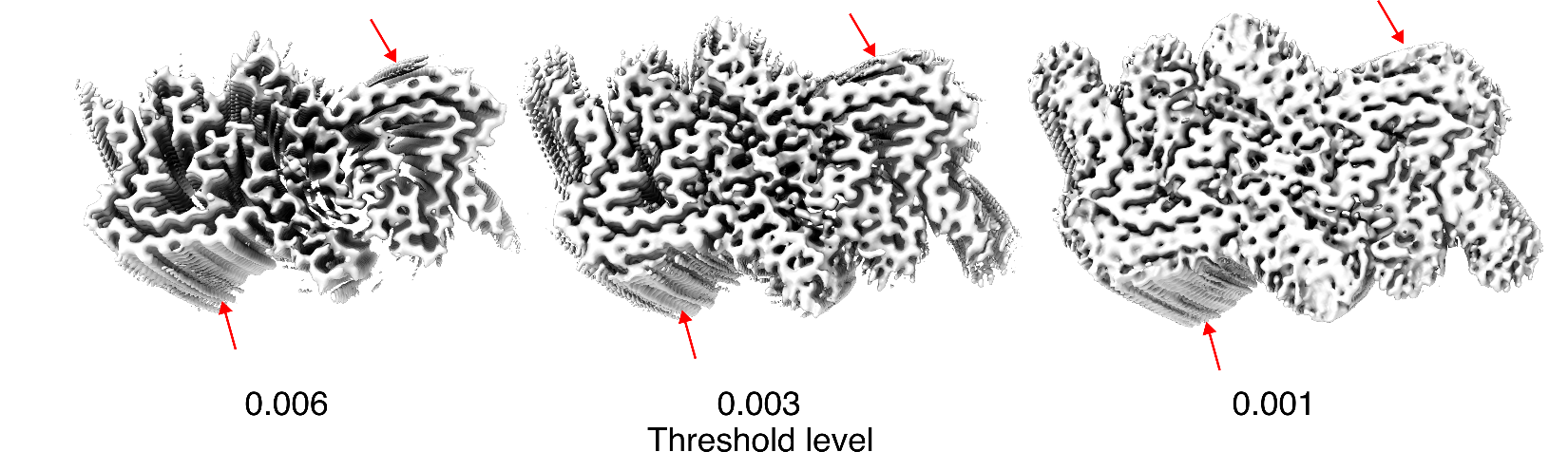


Figure 11. Volume map of aCSF fibrils at different threshold levels, red arrows indicate extra density, which might be from C-terminal β-sheet.


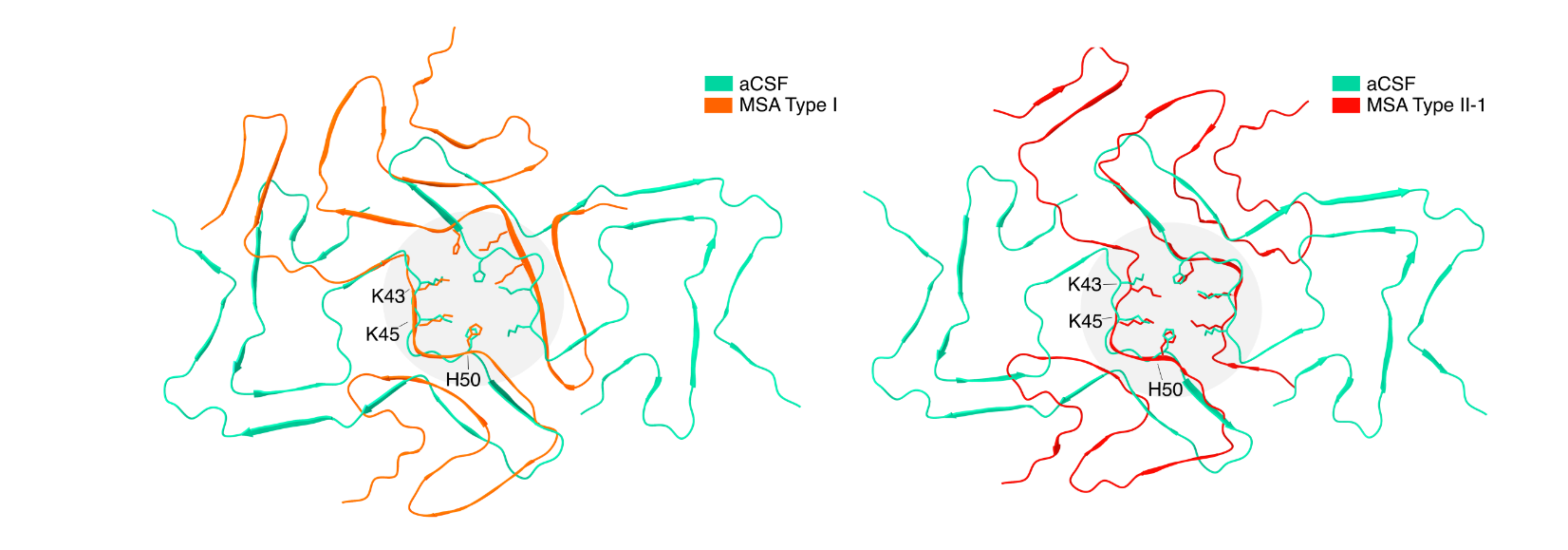


Figure 12. Cryo-EM model comparison between aggregates formed in aCSF and found in patients from MSA (MSA Type I (PDB id: 6XYO) and MSA Type II-1 (PDB id: 6XYP)). For overlay, electron density pocket (K43, K45, H50) was used as a center point.


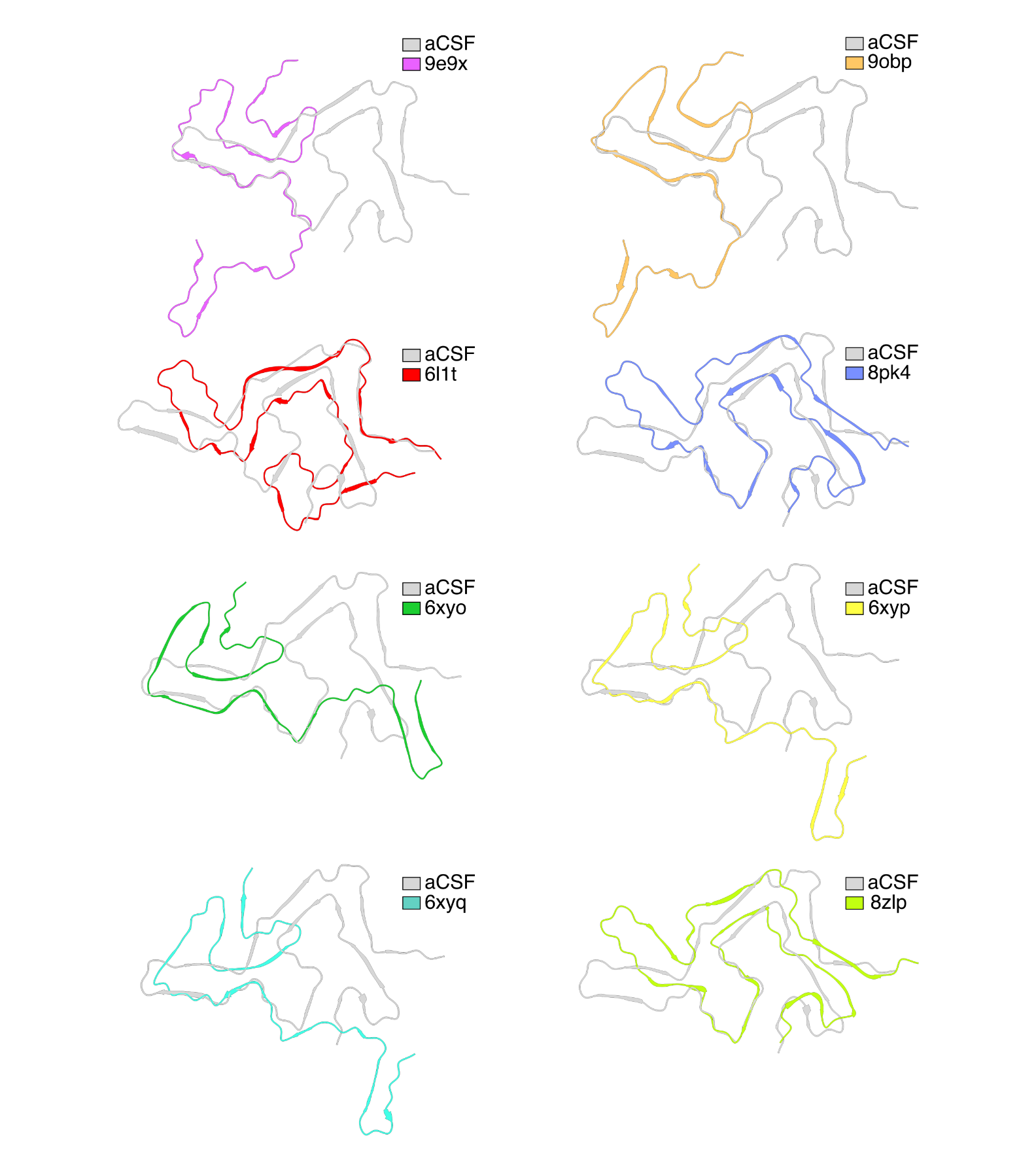


Figure 13. Comparison of different single protofilament structures published online with aCSF filament. The overlay was performed by running a matchmaker (mmaker) command in ChimeraX software.


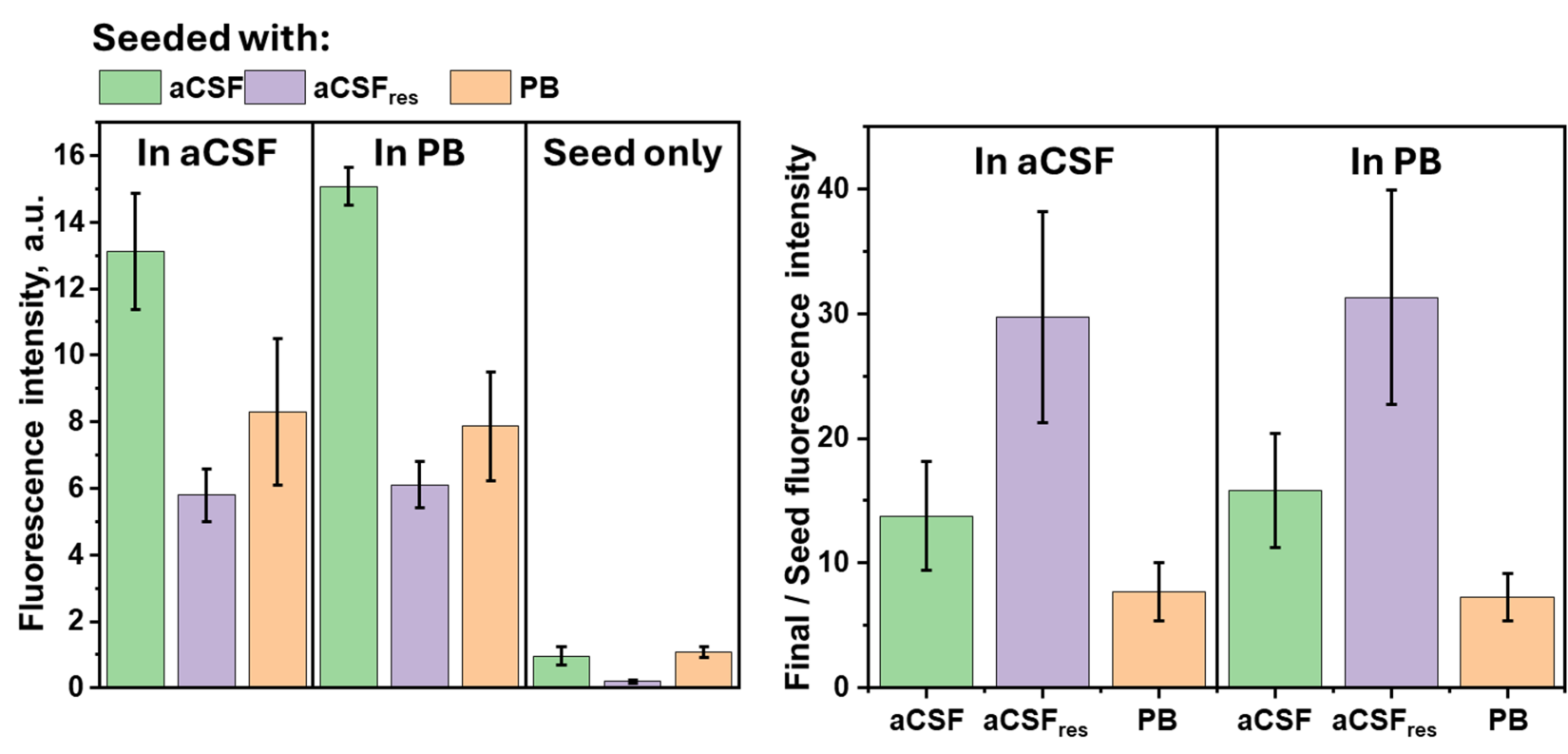


Figure 14. Seeded aggregation kinetics endpoint ThT fluorescence intensity values of aggregation done in aCSF, in PB using 10 % of sonicated seeds formed in both conditions displayed on the left. The ratio between final and seed fluorescence intensities displayed on the right. aCSF_res_ - resuspended aCSF fibrils with PB and incubated (7 days).


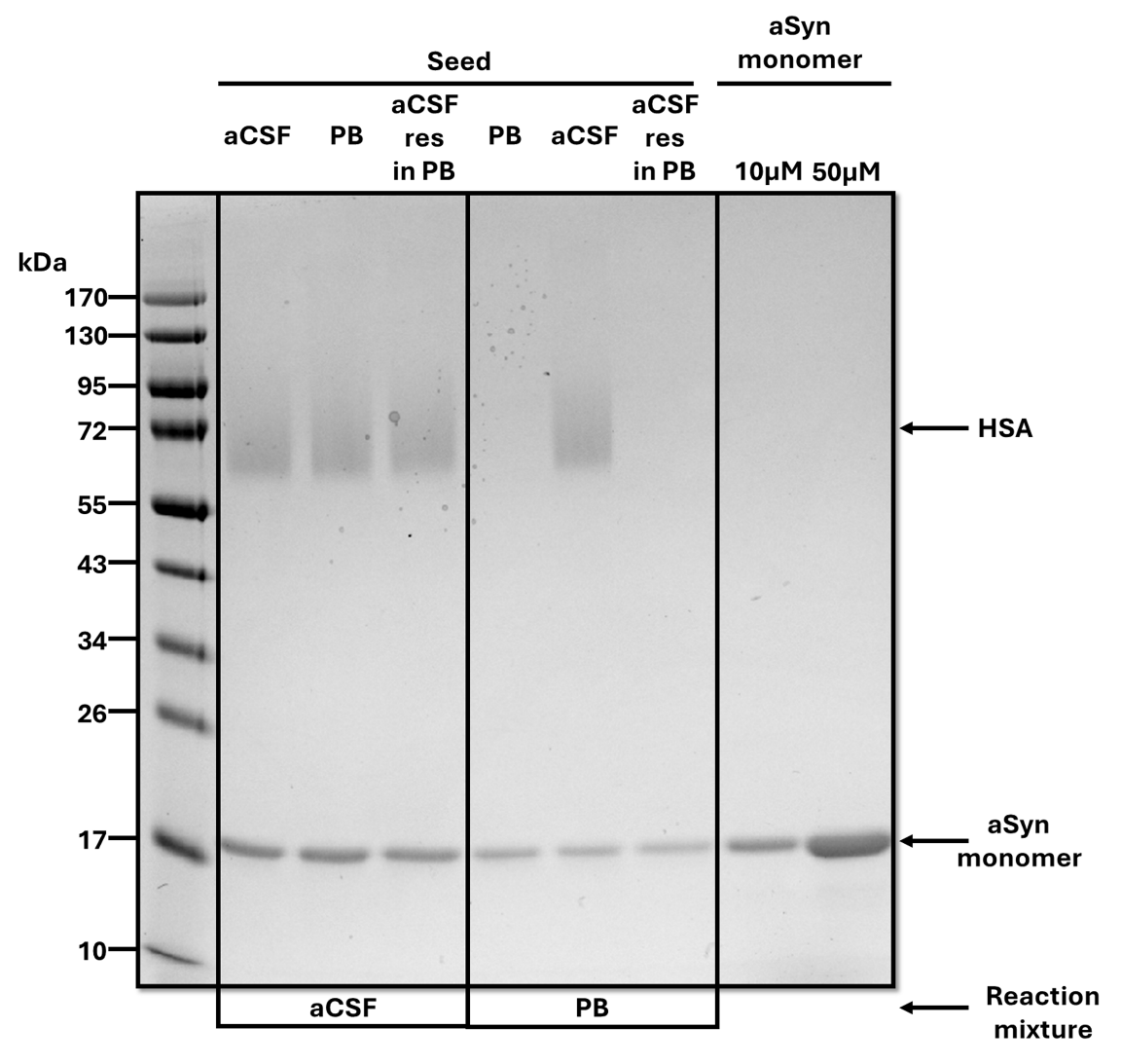


Figure 15. SDS-PAGE of sample supernatants after pelleting final seeded aggregation mixtures. The seeded aggregation was done in aCSF and PB using 10 % of sonicated seeds prepared in aCSF and PB conditions.


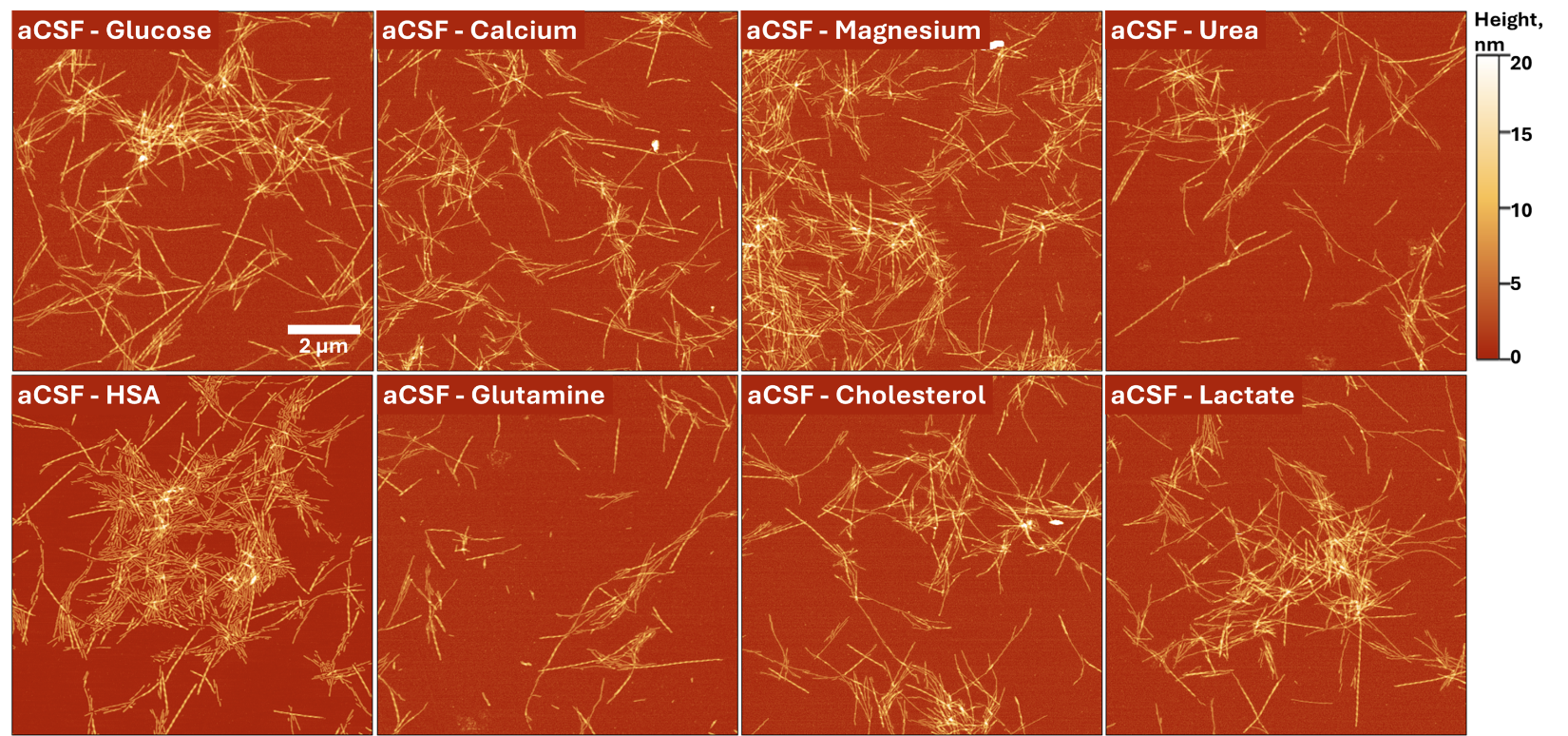


Figure 16. AFM images of aCSF fibrils resuspended in the solution with one of its components removed (indicated top left corner of each picture).


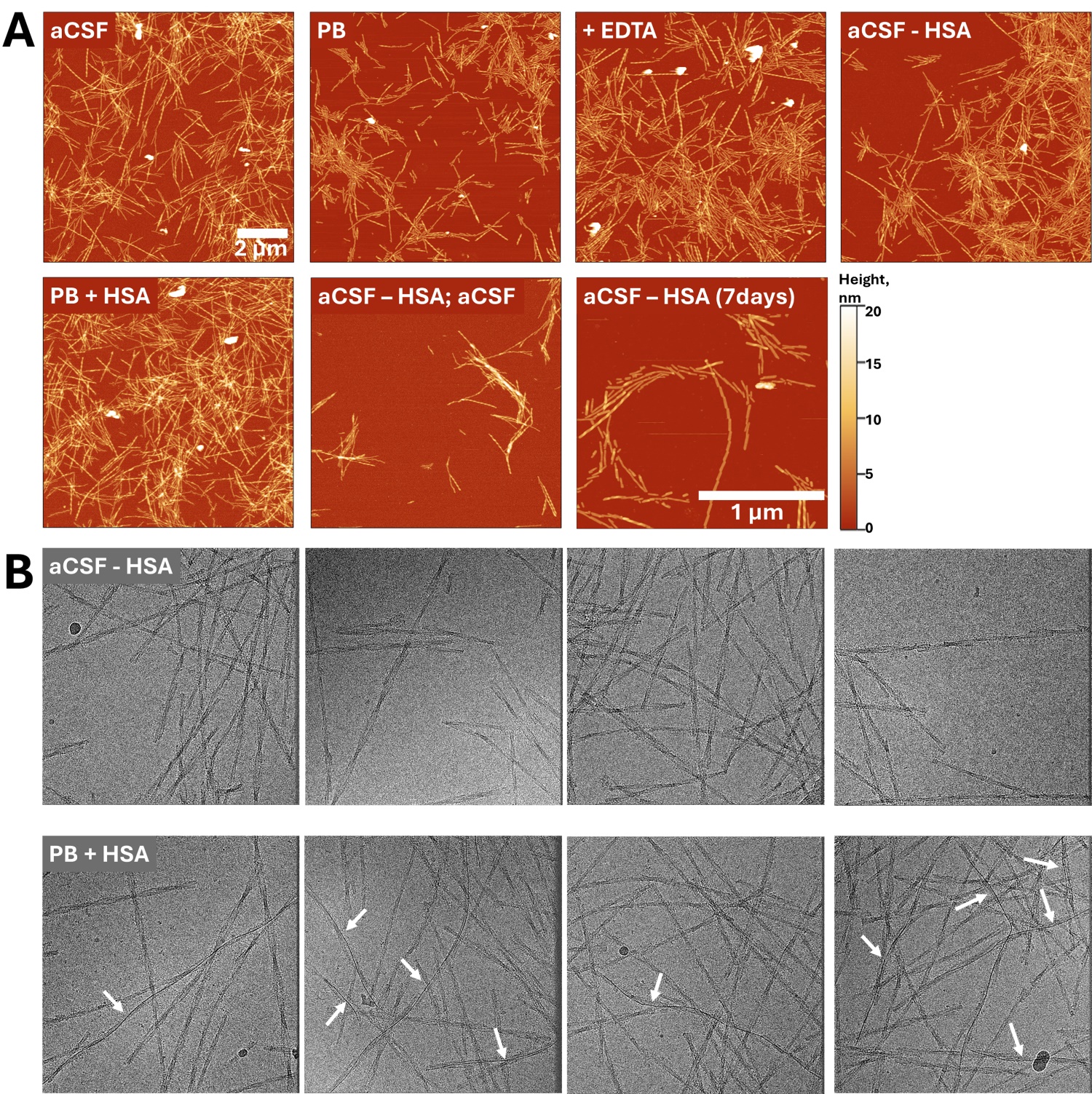


Figure 17. AFM (A) and Cryo-EM (B) images of aCSF fibrils resuspended in different aCSF compositions. In case of aCSF – HSA; aCSF, fibrils were resuspended in aCSF – HSA and then resuspended in aCSF.


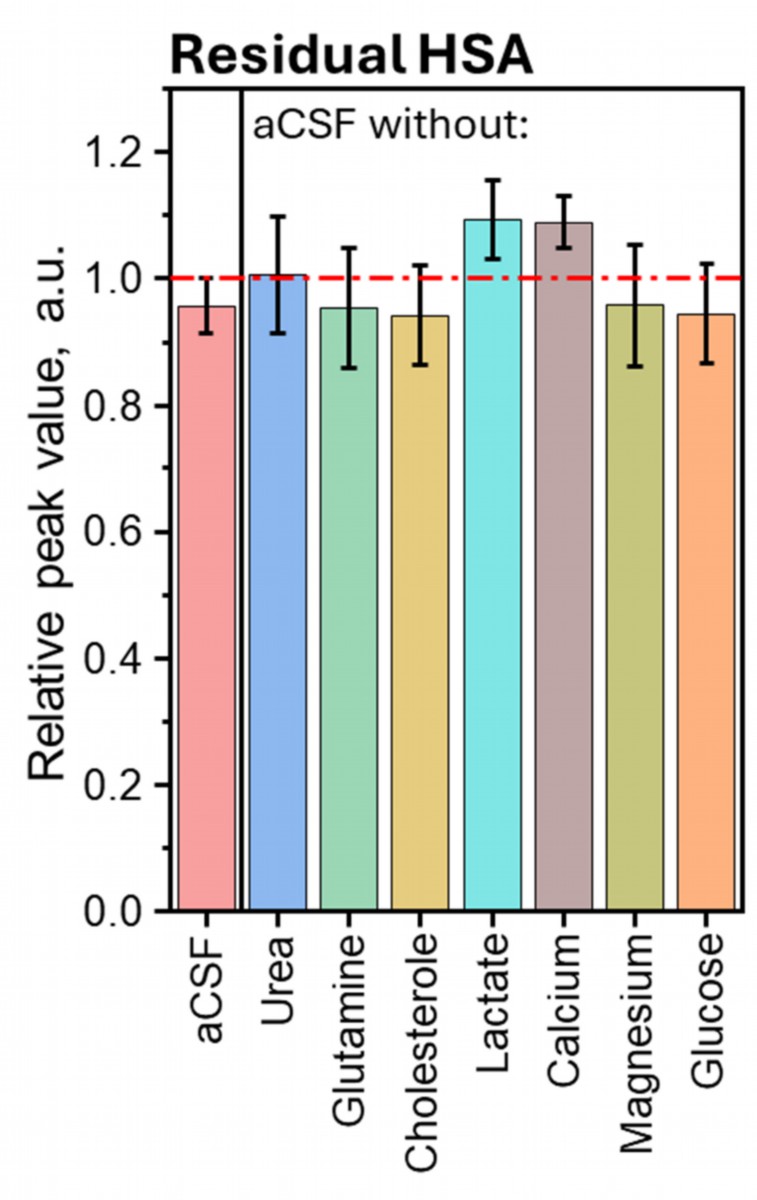


Figure 18. Extracted endpoint concentration of HSA based on SDS-PAGE images using GelAnalyzer 23.1.1 software. The normalization between different SDS-PAGE images was done according to two standard samples with reference to HSA concentration. Eight technical repeats were used for each condition. The error bars are of one standard deviation.


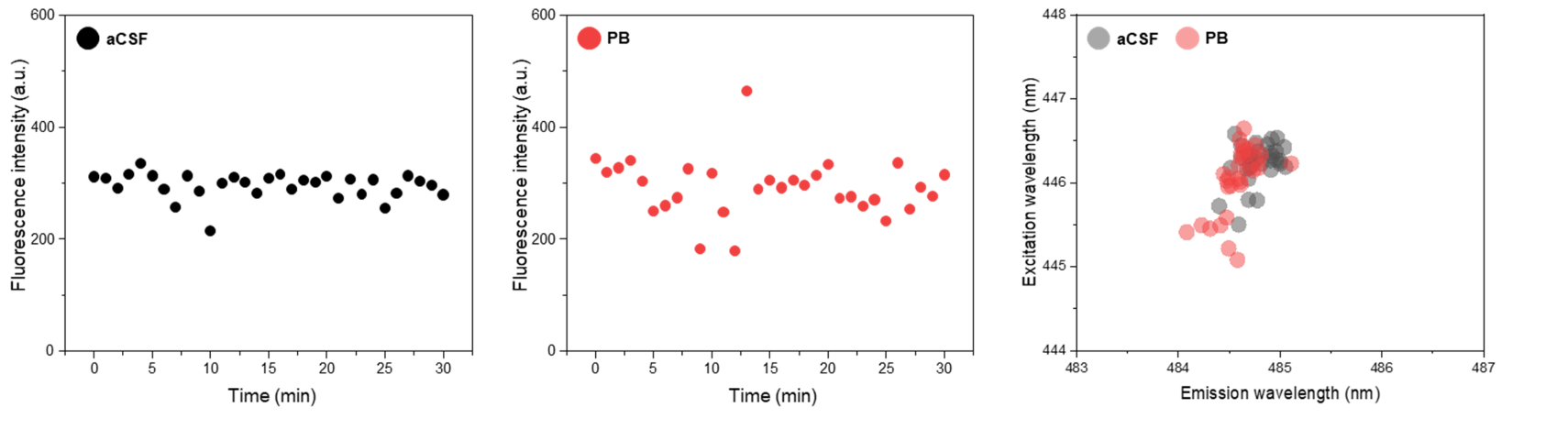


Figure 19. EEM intensity maximum position variation over time of aCSF fibril-bound ThT once they are resuspended in aCSF or PB solutions.


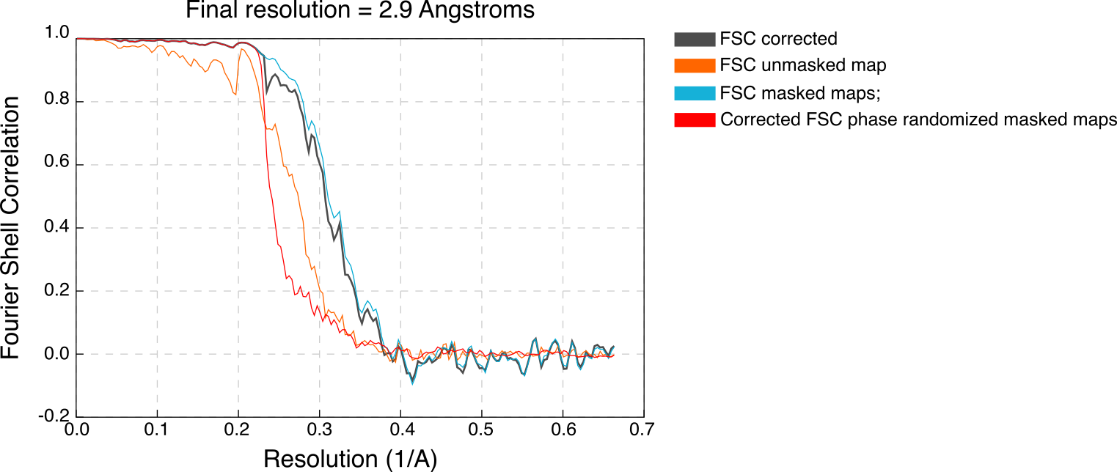


Figure 20. Fourier shell correlation (FSC) curves after final post-processing in Relion.
